# Integration of Canonical and Noncanonical Wnt Signaling Pathways Patterns the Neuroectoderm Along the Anterior–Posterior Axis of Sea Urchin Embryos

**DOI:** 10.1371/journal.pbio.1001467

**Published:** 2013-01-15

**Authors:** Ryan C. Range, Robert C. Angerer, Lynne M. Angerer

**Affiliations:** National Institute of Dental and Craniofacial Research, National Institutes of Health, Bethesda, Maryland, United States of America; German Cancer Research Center, Germany

## Abstract

Patterning the neuroectoderm along the anterior–posterior (AP) axis is a critical event in the early development of deuterostome embryos. However, the mechanisms that regulate the specification and patterning of the neuroectoderm are incompletely understood. Remarkably, the anterior neuroectoderm (ANE) of the deuterostome sea urchin embryo expresses many of the same transcription factors and secreted modulators of Wnt signaling, as does the early vertebrate ANE (forebrain/eye field). Moreover, as is the case in vertebrate embryos, confining the ANE to the anterior end of the embryo requires a Wnt/β-catenin-dependent signaling mechanism. Here we use morpholino- or dominant negative-mediated interference to demonstrate that the early sea urchin embryo integrates information not only from Wnt/β-catenin but also from Wnt/Fzl5/8-JNK and Fzl1/2/7-PKC pathways to provide precise spatiotemporal control of neuroectoderm patterning along its AP axis. Together, through the Wnt1 and Wnt8 ligands, they orchestrate a progressive posterior-to-anterior wave of re-specification that restricts the initial, ubiquitous, maternally specified, ANE regulatory state to the most anterior blastomeres. There, the Wnt receptor antagonist, Dkk1, protects this state through a negative feedback mechanism. Because these different Wnt pathways converge on the same cell fate specification process, our data suggest they may function as integrated components of an interactive Wnt signaling network. Our findings provide strong support for the idea that the sea urchin ANE regulatory state and the mechanisms that position and define its borders represent an ancient regulatory patterning system that was present in the common echinoderm/vertebrate ancestor.

## Abstract 2

Three different Wnt signaling pathways function to restrict the anterior neuroectoderm state to the anterior end of the sea urchin embryo, a mechanism of anterior fate restriction that could be conserved among deuterostomes.

## Author Summary

The initial regulatory state of most cells in many deuterostome embryos, including those of vertebrates and sea urchins, supports anterior neural fate specification. It is important to restrict this neurogenic potential to the anterior end of the embryo during early embryogenesis, but the molecular mechanisms by which this re-specification of posterior fate occurs are incompletely understood in any embryo. The sea urchin embryo is ideally suited to study this process because, in contrast to vertebrates, anterior–posterior neuroectoderm patterning occurs independently of dorsal-ventral axis patterning and takes place before the complex cell movements of gastrulation. In this study, we show that a linked, three-step process involving at least three different Wnt signaling pathways provides precise spatiotemporal restriction of the anterior neuroectoderm regulatory state to the anterior end of the sea urchin embryo. Because these three pathways impinge on the same developmental process, they could be functioning as an integrated Wnt signaling network. Moreover, striking parallels among gene expression patterns and functional studies suggest that this mechanism of anterior fate restriction could be highly conserved among deuterostomes.

## Introduction

Wnt signaling pathways play fundamental roles in many developmental processes. One of the earliest and most crucial of these roles is the activation of gene regulatory programs that specify different cell fates along the embryo's primary anterior–posterior (AP) axis. Recent comparative analyses suggest that Wnt/β-catenin signaling is an ancient AP patterning mechanism that establishes posterior identity in most metazoan embryos [1]–[9]. In invertebrate deuterostome embryos, which include cephalochordates, urochordates, hemichordates, and echinoderms, localized determinants cause stabilization of β-catenin in posterior blastomeres. This stabilized β-catenin enters nuclei in which it activates genes that specify endomesoderm, marking the site of gastrulation at what corresponded to the vegetal pole of the egg and forming the posterior end of the developing embryo [2],[4],[10],[11]. In the sea urchin embryo, in which the molecular mechanisms of endomesoderm specification are best understood [12], the first evidence of Wnt signaling after fertilization is the presence of β-catenin in the nuclei (nβ-catenin) of posterior cells, beginning at the 16- to 32-cell stage. During the next few cleavages, a detectable gradient of nβ-catenin forms in the posterior half of the embryo, with the highest concentration at the posterior pole [4]. This gradient of Wnt/β-catenin signaling is both necessary and sufficient to activate the gene regulatory networks that establish mesoderm and endoderm cell fates in a posterior-to-anterior wave during late cleavage stages [13],[14].

Wnt/β-catenin signaling also transforms the initial regulatory state that specifies anterior neuroectoderm (ANE) development in those deuterostome embryos in which it has been examined [15]–[22]. In the sea urchin embryo, we refer to this neuroectoderm as ANE because it becomes restricted to a region derived from the animal pole of the egg, which is located opposite to the posterior end of the embryo (see [23]). The initial regulatory state of early sea urchin embryos activates ANE specification by the 32-cell stage, when genes encoding the earliest ANE regulatory proteins are expressed broadly throughout the anterior half of the embryo [22],[24]. These early factors include Six3, which is expressed at the anterior end of bilaterian embryos [25] and has been shown by functional studies to be critical for the specification of anterior-most neuroectoderm in diverse embryos including *Tribolium castaneum* [26], sea urchins [24], zebrafish, and mouse [27]. Beginning around the 60-cell stage, a progressive posterior-to-anterior down-regulation of ANE factor gene expression in most of the anterior half of the embryo occurs by an unknown mechanism that requires posterior Wnt/β-catenin signaling [22]. This process continues during blastula stages until the ANE regulatory state is confined to a disk of cells around the anterior pole of the mesenchyme blastula [24]. Interestingly, an unknown signal from posterior Wnt/β-catenin signaling also appears to be necessary to pattern the anterior ectoderm along the AP axis in *Saccoglossus kowalevskii* [2], which belongs to the hemichordates, a sister clade to echinoderms.

**Figure 1:**
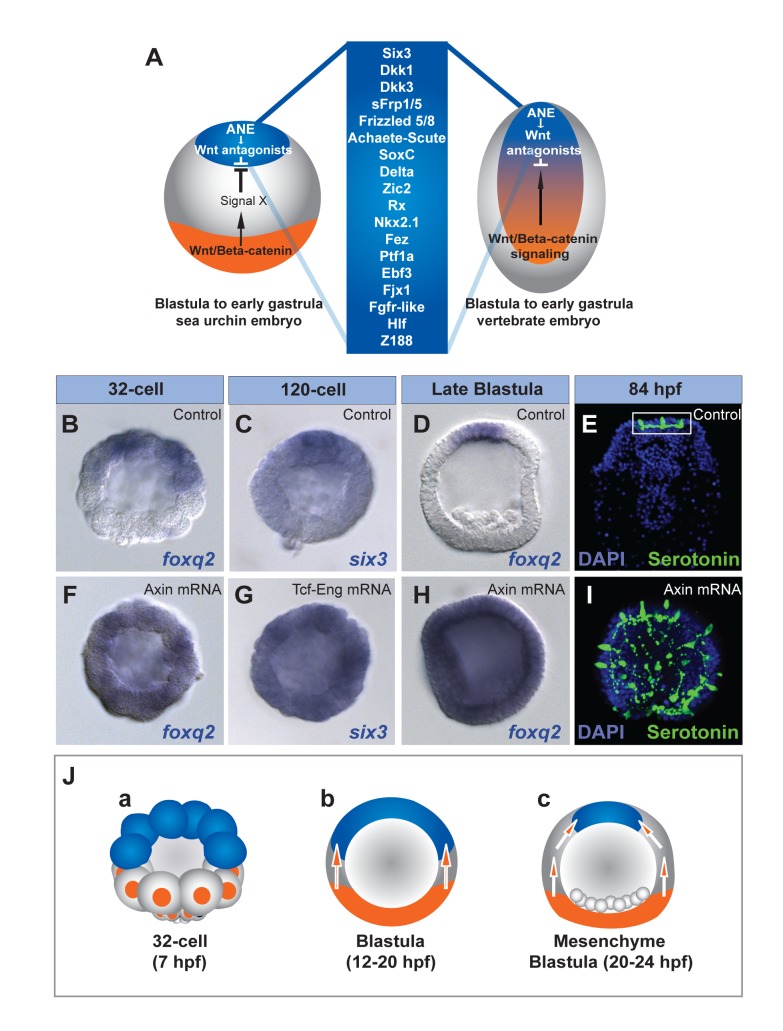
Preventing Wnt/β-catenin signaling allows activation of presumptive ANE specification throughout the embryo. (A) Diagram showing the regulatory factors shared between the sea urchin ANE and the vertebrate forebrain/eye field. Both territories are restricted by mechanisms dependent on posterior Wnt signaling. (B, D, F, H) *foxq2* expression in glycerol control (B, D) or Axin mRNA-injected embryos (F, H) at the 32-cell stage (B, F) and late blastula stage (D, H). *six3* expression in glycerol control (C) and TCF-Eng mRNA-injected early blastulae (G). (E) A normal 3.5-d pluteus stage embryo. (I) A 3.5-d embryo misexpressing Axin mRNA. White box outlines the ANE. Serotonergic neurons (green), DAPI (nuclei, blue). (J) Schematic showing the stages of ANE restriction in 32-cell stage (6 hpf) (a), early blastula stage (7–15 hpf) (b), and mesenchyme blastula stage (24 hpf) embryos (c). ANE (blue), nβ-catenin (orange nuclei at 32-cell stage), endomesoderm territory (orange at blastula stages), posterior ectoderm (gray at blastula stages), and unknown restriction mechanism activated by posterior nβ-catenin (orange arrows).

Remarkably, Six3 activates a large cohort of genes in the sea urchin ANE that are orthologs of genes expressed in the vertebrate ANE (forebrain/eye field) (Figure 1A), raising the possibility that the common ancestor of sea urchins and vertebrates may have shared this ANE regulatory program [24]. Similar to the sea urchin embryo, an initial widespread regulatory state in late blastula/early gastrula stages of vertebrate embryos supports expression of genes encoding early anterior forebrain/eye field factors throughout the presumptive neuroectoderm, including *six3* [28],[29]. Simultaneously, secreted antagonists from the organizer block bone morphogenetic protein (BMP) signaling on the dorsal side and a high-to-low, posterior-to-anterior gradient of nβ-catenin forms in the presumptive neuroectoderm. This Wnt/β-catenin signaling gradient is part of a mechanism that activates genes encoding posterior neuroectoderm factors while down-regulating anterior (forebrain/eye field) factors in the posterior neuroectoderm [16],[18],[21]. By these mechanisms, the neural plate is formed and expression of the presumptive forebrain/eye field factors is restricted to cells at its anterior end, where Wnt antagonists protect them from posteriorization [30]–[33]. Multiple Wnts, Fzl receptors, and Wnt antagonists (i.e., Wnt8, Wnt3a, Wnt1, Fzl8, and Dkk1) have been implicated in posteriorization of the neuroectoderm in vertebrate embryos, as well as members of the fibroblast growth factor (FGF), retinoic acid (RA), and transforming growth factor–beta (TGF-β) signaling pathways [28],[30],[34],[35]. However, the exact functions of these pathways in AP neuroectoderm patterning have been difficult to determine because of their earlier functions as well as the complex cell movements of gastrulation during this process [28],[30],[36]. Moreover, the interactions among these various pathways in AP neuroectoderm patterning are not well understood.

Recent studies suggest that early AP neuroectoderm patterning in vertebrate embryos is independent of information from the dorsal organizer. In both *Xenopus* and zebrafish, the initial widespread regulatory state promotes neuroectoderm specification throughout most of the embryo in the absence of β-catenin, which blocks dorsal organizer formation, as well as BMP2, BMP4, and BMP7. Expression of neuroectoderm markers is radialized around the AP axis in these embryos, but remarkably they retain normal AP neuroectoderm patterning, and the ANE expands posteriorly when both maternal and zygotic Wnt/β-catenin function is blocked [20],[21]. Interestingly, in the sea urchin embryo, the action of Wnt/β-catenin signaling in early patterning of the neuroectoderm along the AP axis [22] is also separate and distinct from the dorsal-ventral patterning mechanism because it occurs before Nodal and BMP signaling are activated and is required for their expression [37]–[40]. The inhibition of Wnt/β-catenin signaling, and consequently the loss of expression of Nodal and BMP, causes a large majority of cells to differentiate into ANE [22],[24],[41]. Thus, the developmental regulatory mechanisms used by vertebrate embryos for ANE development have striking similarities to those used by sea urchin embryos and may therefore represent an ancestral deuterostome mechanism.

Here we show that the Wnt-dependent restriction of neuroectoderm to the anterior pole involves not only Wnt/β-catenin but also a series of linked steps mediated by Wnt/JNK signaling through Wnt1, Wnt8, and Fzl5/8, the homolog of vertebrate Fzl8. Coordinated progression of signaling through these Wnt pathways and activation of the secreted Wnt antagonist Dkk1 in anterior-most blastomeres establish the definitive ANE around the anterior pole. Signaling through a second Wnt receptor, Fzl1/2/7, and its activation of PKC suppress Wnt/β-catenin and Wnt/JNK ANE restriction activities to coordinate the correct temporal progression of ANE restriction. Collectively, signaling through three different Wnt signaling pathways provides precise spatiotemporal control of neuroectoderm AP patterning along the AP axis.

## Results

### Wnt/β-Catenin Signaling Prevents ANE Specification in Posterior Blastomeres

FoxQ2 and Six3 are essential for the specification of the ANE and are the earliest ANE regulatory genes to be expressed. Their transcripts accumulate in the anterior half of the 32-cell embryo but are never detectable in the posterior half (Figure 1B,C) [22],[24]. We reasoned that posterior repression might depend on Wnt/β-catenin signaling because this pathway is activated in posterior blastomeres by the 16-cell stage [4],[14],[42],[43]. To test this possibility, we blocked nβ-catenin by injecting embryos with mRNA encoding either Tcf-Engrailed (Tcf-Eng) [42] or Axin [44] and examined *foxq2* expression at the 32-cell stage (Figure 1B,F; Figure S1). In both cases, *foxq2* and *six3* were expressed in every blastomere during early cleavage stages (32-cell, Figure 1F; 120-cell, Figure 1G) and ubiquitous expression persisted until late mesenchyme blastula stage (24 hpf) (Figures 1G,H and S1Ae–g). As expected, each perturbation resulted in formation of dauer blastulae with a thickened neuroepithelium covering most of the embryo that produced greatly increased numbers of serotonergic neurons throughout (Figure 1I versus E; Figure S1Ah versus Ad). These 4-d embryos phenocopied ΔCadherin mRNA-injected embryos, which previously were shown to lack nβ-catenin in all but the four vegetal-most blastomeres, the small micromeres during cleavage stages [24]. Together, these data indicate that the factors that activate ANE specification operate in all early blastomeres in these Wnt/β-catenin-deficient embryos and likely are part of a ubiquitous maternal regulatory state. Moreover, these observations indicate that the first step in suppressing the ANE in the posterior half of the embryo depends on the repression or rapid down-regulation of ANE regulatory gene transcription by Wnt/β-catenin signaling.

### Fzl5/8 Signaling and JNK Activity Are Required to Down-Regulate the ANE Regulatory State in Posterior Ectoderm Cells

**Figure 2:**
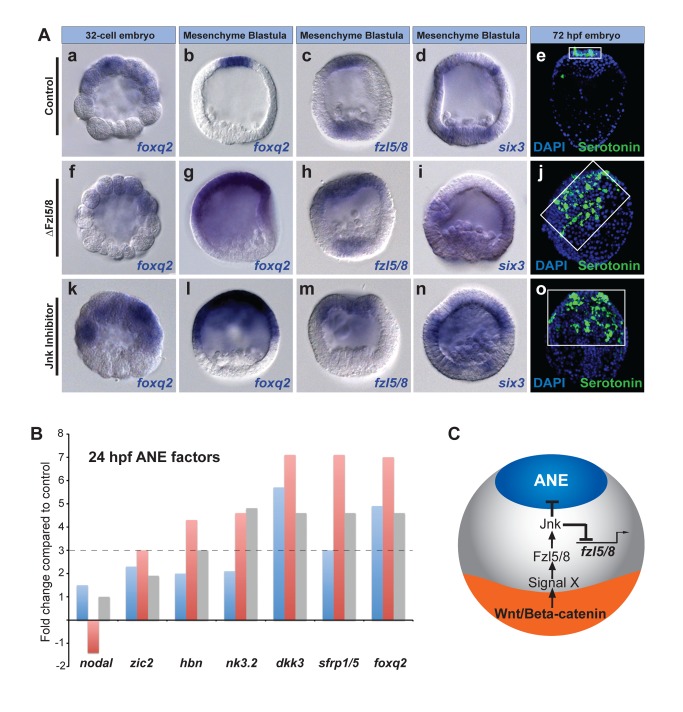
Fzl5/8 signaling and JNK activity are required for ANE restriction. (A) *foxq2*, *fzl5/8*, and *six3* expression in 32-cell and late blastula-stage control embryos (Aa–d), ΔFzl5/8 mRNA-injected embryos (Af–i), and embryos treated with JNK inhibitor (Ak–n). (Ae, j, o) Serotonergic neurons in control, ΔFzl5/8 mRNA-injected, and JNK inhibitor-treated embryos, respectively. White boxes outline the ANE. Serotonergic neurons (green), DAPI (nuclei, blue). (B) qPCR measurements from three different cultures of embryos showing that ANE regulatory genes are up-regulated at the late blastula stage (24 hpf) in the absence of functional Fzl5/8 signaling. The *y*-axis shows the fold change in gene expression in ΔFzl5/8-containing embryos relative to controls. The dotted line marks a 3-fold change in expression. *nodal* expression is used as an internal control because it is unaffected in the absence of Fzl5/8 signaling (Croce et al., 2006) [46]. (C) Diagram showing a model for ANE restriction consistent with the data presented in this figure.

Previous studies have shown that restriction of *foxq2* expression to the anterior pole depends on posterior Wnt/β-catenin signaling. However, Wnt/β-catenin signaling has never been detected in the anterior half of the embryo (the presumptive ectoderm, blue in Figure 1Ja), suggesting that an intermediate signal(s) downstream of posterior Wnt/β-catenin signaling must mediate this second phase of ANE restriction (Figure 1Jb versus Jc; the gray region in this and subsequent figures represents the posterior ectoderm and the orange arrows indicate the second phase of restriction). We hypothesized that this intermediate signal (Figure 2C, signal X) might also involve Wnt signaling, and we tested this idea by exploring the functions of the Wnt [Frizzled (Fzl)] receptors in ANE restriction. Two of the four sea urchin receptors, Fzl5/8 and Fzl1/2/7, were expressed during ANE restriction (Figure S2A) and also in the appropriate cells to mediate this process (Figure S2Ba–h), making them excellent candidates for transducing Wnt signals that eliminate the ANE regulatory state from the posterior ectoderm (Figure S2Bi–Bl).

To determine whether Fzl5/8 signaling has a role in neuroectoderm AP patterning, we injected embryos either with morpholinos targeting Fzl5/8 or with mRNA encoding a C-terminal truncated form of the receptor (ΔFzl5/8) that acts as a dominant negative by competing for Wnt ligands [45]. In contrast to embryos injected with Axin or Tcf-Eng mRNA, those expressing ΔFzl5/8 mRNA had normal *foxq2* transcript levels and distributions at the 32-cell stage (cf., Figure 2Aa,Af), suggesting that Fzl5/8 signaling is not required for the initial Wnt/β-catenin-dependent down-regulation of *foxq2* mRNA in the posterior half of the embryo. Further evidence that Fzl5/8 is not required for early Wnt/β-catenin activity is provided below. However, at mesenchyme blastula stage (24 hpf), ΔFzl5/8-injected embryos expressed *foxq2* ectopically throughout the anterior half of the embryo, indicating that the second phase of its restriction to the anterior pole requires Fzl5/8 function (cf., Figure 2Ab,Ag). Expression of *foxq2* also was not correctly restricted in two different Fzl5/8 morphants, although the phenotype was less pronounced (cf., Figure 2Ab,Ag versus Figure S3D,E). We used ΔFzl5/8 for further studies because it gave the more penetrant phenotype, likely because it blocked signaling through both maternal and zygotic Fzl5/8. Importantly, eliminating expression of *six3*, the critical upstream ANE regulator, from the posterior ectoderm also required functional Fzl5/8 signaling (Figure 2Ad,Ai). Furthermore, the transcript levels per embryo for genes in the 24 hpf Six3-dependent ANE regulatory network (Figure 2B) were significantly elevated in ΔFzl5/8-containing mesenchyme blastula embryos. Interestingly, one of these was zygotic *fzl5/8* mRNA itself (Figure 2Ac,Ah), indicating that Fzl5/8 function is required to down-regulate *fzl5/8* mRNA levels in the posterior ectoderm. Finally, 3-d pluteus larvae injected with ΔFzl5/8 had an expanded thick neuroepithelium with a greatly increased number of serotonergic neurons (Figure 2Aj). In contrast, the thickened neuroepithelium in normal pluteus-stage embryos was restricted to a small region that produced only 4–6 serotonergic neurons (Figure 2Ae). These observations indicate that a Fzl5/8 signaling-dependent process eliminates the ANE regulatory state required for serotonergic neural development from the posterior ectoderm (Figure 2C).

In addition to the ubiquitous maternal and anterior zygotic expression of *fzl5/8* at mesenchyme blastula stage (Figure S2e–h), it was also expressed in a ring of nonskeletogenic mesenchyme cells (24 hpf) (Figures 2Ac and S2Bh). Previously, Croce et al. (2006) [46] showed that Fzl5/8 signaling in these posterior cells works through the c-Jun N-terminal kinase (JNK) pathway to initiate primary invagination movements later during gastrulation. This observation raised the possibility that the earlier ANE restriction process mediated by Fzl5/8 in posterior ectoderm may also depend on the JNK pathway. *jnk* mRNA was present ubiquitously during ANE restriction (Figure S2C), and indeed, *foxq2* failed to restrict to the anterior pole in embryos injected with a splice-blocking JNK morpholino (Figure S3A). This JNK morphant phenotype was weaker than the ΔFzl5/8 phenotype (cf., Figure 2G and Figure S3A), probably because some normal JNK transcripts persisted in the embryo (Figure S3J). It is also possible that maternally synthesized JNK protein persisted in these embryos. As an additional test, we treated embryos with the specific JNK inhibitor, (L)-JNKI1 [46],[47], beginning at fertilization, which produced embryos expressing *foxq2* throughout the anterior half of the embryo, mimicking exactly the ΔFzl5/8 phenotype (Figure 2, cf. Ag,Al). Moreover, *fzl5/8* and *six3* expression was not restricted to the anterior pole (Figure 2Am,An), and these embryos also had an expanded, thickened neuroepithelium and an increased number of serotonergic neurons, as seen in ΔFzl5/8-injected embryos (Figure 2Aj). These results indicate that the second phase of ANE restriction that down-regulates the ANE regulatory state in the anterior half (i.e., the posterior ectoderm) depends on Fzl5/8 function. Moreover, they suggest that JNK activity transduces a Wnt signal X through this Wnt receptor, the production of which depends on Wnt/β-catenin activity in the posterior half of the embryo (signal X, Figure 2C).

### Wnt1 and Wnt8 Signals Restrict the ANE to the Anterior Pole

**Figure 3:**
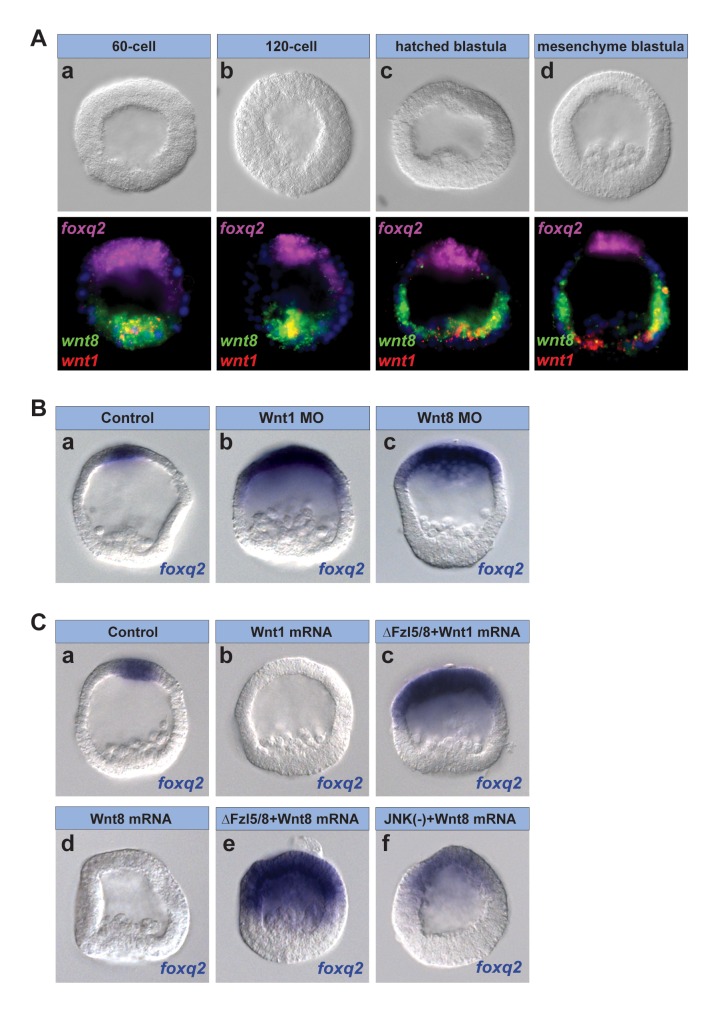
Wnt1 and Wnt8 signaling are necessary for Fzl5/8-JNK-mediated ANE restriction. (A) Three-color in situ hybridization for *wnt1* (red), *wnt8* (green), and *foxq2* (magenta) transcripts during ANE restriction. *wnt1 mRNA* appears yellow when overlapping with *wnt8 mRNA*. (B) The *foxq2* expression domain is not restricted in embryos injected with a Wnt1 (b) or Wnt8 (c) morpholino. (C) *foxq2* expression is completely eliminated in embryos injected with either Wnt1 (b) or Wnt8 (d) mRNA. The Wnt1- and Wnt8-mediated inhibition of *foxq2* expression requires functional Fzl5/8 (c, e); the Wnt8-mediated inhibition of *foxq2* expression requires JNK activity (f). MO, morpholino.

To identify the link between Wnt/β-catenin signaling and Fzl5/8, we first searched for genes encoding Wnt ligands that are expressed by the 60-cell stage (i.e., the beginning of the second phase of ANE restriction) in posterior blastomeres and that also depend on Wnt/β-catenin activity. We confirmed the previously reported expression profile of *wnt8*, which is activated by Wnt/β-catenin [48],[49]: At the 60-cell stage, *wnt8* was expressed in both the micromeres and the adjacent blastomere tier (veg_2_) (Figure 3Aa). Similarly, *wnt1* expression was first detected midway through the 60-cell stage (9 hpf) in the micromeres, and it also depended on nβ-catenin (Figures 3Aa and S4A,C). As development progressed, *wnt8* expression first moved into the next most anterior tier of blastomeres (veg_1_) and then, during late blastula stages (18 and 24 hpf), into both veg_1_ and overlying posterior ectoderm cells (Figure 3Ac,Ad). *wnt1* expression continued in the micromeres until midblastula stages (15 hpf) after which it, too, progressively moved to more anterior blastomeres until it reached the endoderm/ectoderm boundary during later blastula stages (24 hpf) (Figures 3Ac,Ad and S4A). Thus, genes encoding the secreted ligands Wnt1 and Wnt8 were expressed in posterior cells when the second phase of ANE restriction begins in the posterior regions of the anterior hemisphere. As restriction proceeded, *wnt8* continued to be expressed near cells expressing ANE marker genes, whereas *wnt1* expression was more posterior. In order to evaluate whether these secreted ligands were required for ANE restriction in posterior ectoderm, we performed knockdown experiments by injecting either of two different morpholinos designed against each. As shown in Figure 3B, embryos injected with either Wnt1 or Wnt8 morpholinos failed to down-regulate *foxq2* expression in posterior ectoderm. ANE restriction was more strongly perturbed in Wnt1 morphants, even though the cells producing it were more distant from the site of action than those producing Wnt8. This raised the possibility that Wnt1 is necessary for later Wnt8 expression. However, this was not the case because, at blastula stage (16 hpf), Wnt8 expression was normal in Wnt1 morphants (Figure S4D). The converse was also true: *wnt1* expression did not depend on Wnt8 (Figure S4E). We conclude that production of each of these ligands depends on Wnt/β-catenin signaling, but they do not depend on each other but act in parallel in ANE restriction.

These results suggest that Wnt1 and Wnt8 spatiotemporally link posterior Wnt/β-catenin signaling to Fzl5/8-mediated down-regulation of ANE factors in the posterior ectoderm. To test this hypothesis, we first showed that overexpressed Wnt1 or Wnt8 completely eliminated *foxq2* expression in the ANE (Figure 3Cb,Cd). We then tested whether this *foxq2* down-regulation required active Fzl5/8. Strikingly, ΔFzl5/8 strongly blocked the suppression of *foxq2* expression mediated by either Wnt1 or Wnt8 (100% rescue of Wnt1 or Wnt8 misexpression phenotype; *n* = 63 and 67, respectively) (Figures 3Cc,Ce and S5Bb,Bd). These results strongly support the conclusion from the Wnt1 and Wnt8 loss-of-function analyses that Fzl5/8-mediated ANE restriction in the posterior ectoderm requires these ligands. Furthermore, suppression of *foxq2* expression by both Wnt 1 and Wnt8 also required JNK activity, since the JNK inhibitor rescued the loss-of-ANE phenotypes produced by misexpression of Wnt8 (81% of embryos rescued; *n* = 126)(Figure 3Ca versus Cf) and, to a lesser extent, Wnt1 (55% of embryos had low to normal *foxq2* expression; *n* = 83) (Figure S5A). These data indicate that Wnt1, Wnt8, and Fzl5/8 function in a Wnt/JNK signaling pathway to effect the second phase of ANE restriction.

### Fzl1/2/7 Signaling and PKC Activity Are Necessary for ANE Specification

**Figure 4:**
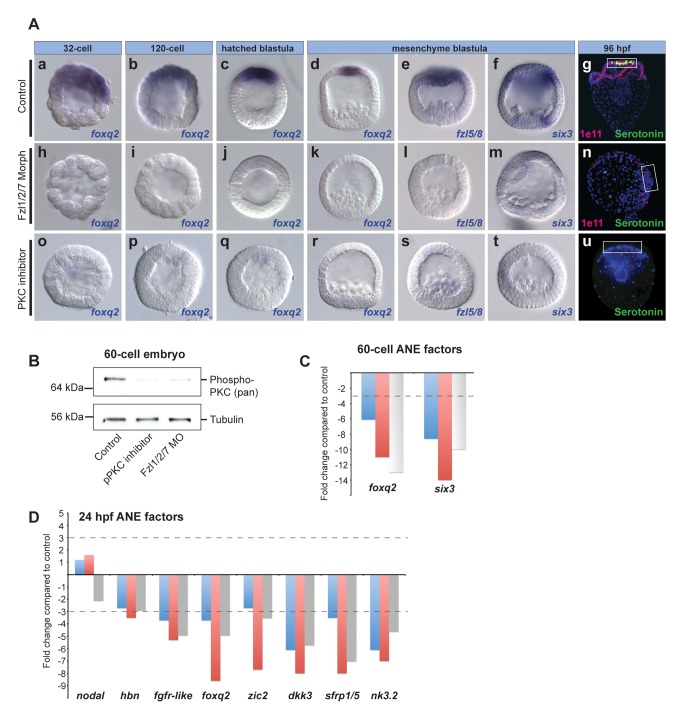
Fzl1/2/7 signaling and PKC activity are necessary for ANE specification. (A) *foxq2* expression at stages from 32-cell to hatched blastula, as well as *fzl5/8* and *six3* expression at mesenchyme blastula stage in control embryos (Aa–f), Fzl1/2/7 morpholino-injected embryos (Ah–m), and embryos treated with PKC inhibitor (Ao–t). (Ag, n, u) Neurons in control, Fzl1/2/7 morpholino-injected, and PKC inhibitor-treated 96-hpf pluteus larvae. White boxes outline the ANE. Serotonergic neurons (green), DAPI (nuclei, blue), synaptotagminB/1e11 (pan-neural, magenta). (B) Western blot showing that phosphorylation of PKC is blocked to similar extents with a PKC inhibitor and Fzl1/2/7 MO. (C, D) qPCR measurements from three different cultures of embryos showing that the early ANE regulatory genes are down-regulated in the absence of Fzl1/2/7 signaling at the 60-cell stage (C) and late blastula stages (D). The *y*-axis shows the fold change in gene expression level in Fzl1/2/7 morpholino-containing embryos relative to controls. The dotted line marks a 3-fold change in expression. *nodal* expression is used as an internal control.

The expression pattern of the gene encoding the other early Wnt receptor, *fzl1/2/7*, suggests that Fzl1/2/7 signaling also could affect neuroectoderm restriction (Figure S2Ba–Bd). We tested this possibility by morpholino knockdown. We were surprised to find that neither *six3* nor *foxq2* was activated at the 32- to 60-cell stage (Figure 4Aa versus Ah and Figure 4C) and neither mRNA was detectable throughout the normal time of ANE restriction (Figure 4Ah–k, Af- versus Am). As expected, zygotic *fzl5/8* expression, which depends on Six3 [24], also required Fzl1/2/7 (Figure 4Ae versus Al). As well, the expression of all other known regulatory factors that depend on Six3 at mesenchyme blastula stage (24 hpf) also required Fz1/2/7 function (Figure 4D). Moreover, the ectoderm in 3- to 4-d Fzl1/2/7 morphants lacked a thickened columnar epithelium corresponding to the ANE in normal embryos (Figure S3F). In 4-d pluteus larvae, which normally have well-established neurons in the ANE, the large majority of Fzl1/2/7 morphants had none (37/41 embryos) (Figure 4Ag versus An, green). They also had a severely reduced number of ciliary band neurons, as assayed by the pan-neural marker SynaptotagminB (Figure 4An, 1e11 antibody, magenta). These results indicate that Fzl1/2/7-mediated signaling is essential for establishment and maintenance of the early neuroectoderm regulatory state, which in turn subsequently is required for the specification and differentiation of all neurons (Figure 4Ag).

The Fzl1/2/7 morphant phenotype is opposite to the Axin or Tcf-Eng misexpression phenotypes as well as those produced by ΔFzl5/8 misexpression or treatment with the JNK inhibitor or JNK morpholino. These observations raise the possibility that Fzl1/2/7 transduces a different Wnt signal, possibly through the Ca^2+^ pathway. Although the architecture of the Ca^2+^ pathway downstream of Fzl receptors is not yet well established, one important player in other systems is conventional Protein Kinase C (PKC) [50],[51]. In the sea urchin embryo, genes encoding conventional PKC isoforms are expressed maternally and throughout development and at least one is activated by the 60-cell stage (Figure 4B) [52]. To test the hypothesis that pPKC, like Fzl1/2/7, is necessary for maintaining ANE specification, we treated embryos with the specific PKC inhibitor, Bisindolylmaleimide 1, which blocks activation through phosphorylation of most Ca^2+^-dependent PKC isoforms by competing for the ATP binding site [53]. Treatment with this inhibitor at 1–3 µM strongly reduced the level of pPKC (Figure 4B), but had no detectable deleterious effects on the morphology of embryos during ANE restriction. Importantly, the level of pPKC in Fzl1/2/7 morphants was as low as that produced by the PKC-specific inhibitor (Figure 4B), indicating that Fzl1/2/7 function is required for activation of this kinase. Similar to Fzl1/2/7 morphants, *foxq2* expression was never initiated in embryos treated with the inhibitor continuously from fertilization to mesenchyme blastula stage (24 hpf) (Figure 4Ao–r). Moreover, *six3* and *fzl5/8* were not expressed (Figure 4As,At), and in a large majority of embryos (36/39) serotonergic neurons did not develop (Figure 4Au and Figure S3Ia versus Ic), showing that neural differentiation was severely compromised in treated embryos. While these experiments demonstrate that activation of PKC is required for the ANE regulatory state and that Fzl1/2/7 is required for that activation, they do not conclusively prove that Fzl1/2/7 signals through the Ca^2+^ pathway because PKC activation can occur by other mechanisms. We conclude that Fzl1/2/7 signaling and PKC activity are each essential for early neuroectoderm specification.

### Fzl1/2/7 Signaling and PKC Activity Antagonize the ANE Restriction Mechanism

**Figure 5:**
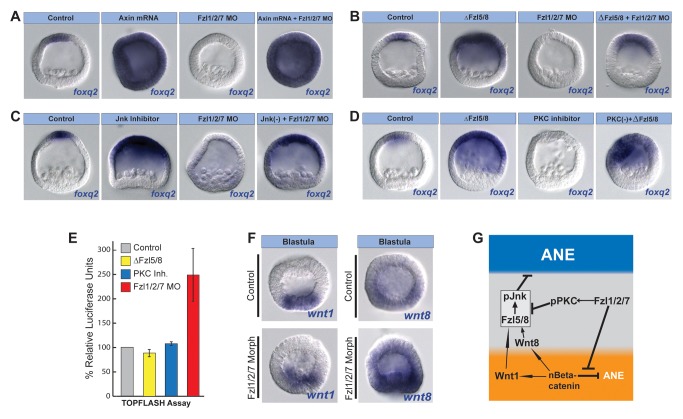
Fzl1/2/7 signaling and PKC activity antagonize the ANE restriction mechanism. (A–D) Injected molecules are indicated above each panel. Inhibition of Fzl1/2/7 function (Fzl1/2/7 MO) does not interfere with *foxq2* expression in the absence of nβ-catenin (Axin mRNA) (A) or functional Fzl5/8 (ΔFzl5/8 mRNA) (B) or when JNK was inhibited (C). (D) Blocking PKC activity in the absence of ΔFzl5/8 signaling rescues *foxq2* expression. (E) TopFlash assays on three different cultures of embryos showing that the activity of Fzl1/2/7, but not those of Fzl5/8 or PKC, suppress nβ-catenin activity. (F) *wnt1* and *wnt8* are expressed in the endomesoderm of Fzl1/2/7 morphants. (G) Diagram showing a model for ANE restriction based on the data presented in this and the preceding figures. MO, morpholino.

Our findings that a Wnt signaling branch utilizing Fzl1/2/7 and PKC activity is necessary for *initiating expression* of upstream ANE regulatory factors was entirely unexpected because at early stages, Wnt signaling is thought to antagonize this process. We hypothesized that Wnt signaling through this receptor is necessary either for the expression of regulatory genes that specify the ANE or for antagonizing the ANE restriction mechanism from the very earliest stages. To distinguish between these alternatives, we first asked whether Fzl1/2/7 signaling is part of the maternal mechanism that can drive ubiquitous expression of ANE regulatory genes in the absence of Wnt/β-catenin signaling. Within each of three batches of embryos, we injected one set of fertilized eggs with Axin mRNA, a second set with Fzl1/2/7 morpholino, and a third with both Fzl1/2/7 morpholino and Axin mRNA (Figure 5A). As shown above, *foxq2* was expressed throughout the embryo in the absence of nβ-catenin, whereas it was completely undetectable in more than 90% (52/57) of embryos lacking Fzl1/2/7. However, it was expressed at high levels throughout all Fzl1/2/7-deficient embryos (47/47) when Wnt/β-catenin signaling was also blocked. These results indicate that maternal factors are still capable of activating *foxq2* in embryos lacking Fzl1/2/7 and that the loss of *foxq2*/ANE fate in Fzl1/2/7 morphants requires a functional Wnt/β-catenin pathway. Thus, Fzl1/2/7 signaling is not a positive regulator of the initial maternal regulatory state that supports ANE specification, but rather it inhibits the Wnt/β-catenin-dependent ANE restriction mechanism.

To test if Fzl1/2/7 also antagonizes the Fzl5/8-JNK-dependent second phase of ANE restriction, we asked whether blocking Fzl5/8 or JNK function could rescue ANE specification in embryos lacking Fzl1/2/7 signaling (Figure 5B,C). Similar to the above experiments, in three different batches of embryos, we found that blocking the function of either Fzl5/8 or the JNK pathway rescued the expanded expression of *foxq2* in 99% (*n* = 72) or 93% (*n* = 70), respectively, of embryos also lacking Fzl1/2/7. These results suggest that Fzl1/2/7 antagonizes Fzl5/8-JNK-mediated ANE restriction. In the final set of experiments, we tested whether PKC signaling also antagonizes Fzl5/8-JNK-mediated ANE restriction (Figure 5D). Using the same approach, we injected one set of fertilized eggs with ΔFzl5/8, treated a second with the PKC inhibitor, and a third was treated with PKC inhibitor and injected with ΔFzl5/8. Blocking the function of Fzl5/8 in these embryos rescued the expression of *foxq2* in a large majority of embryos (77% rescue; *n* = 83), demonstrating that, like Fzl1/2/7, PKC antagonizes the ANE restriction mechanism by antagonizing Fzl5/8 signaling. Collectively, these results support the idea that the Fzl1/2/7-dependent suppression of Fzl5/8-mediated ANE restriction works through PKC (Figure 5G).

The data suggest that Fzl1/2/7 signaling antagonizes Fzl5/8-JNK-mediated down regulation of genes necessary for ANE specification. Because Fzl1/2/7 functions as early as the 32-cell stage to maintain expression of ANE markers, it might also antagonize Fz5/8 indirectly by down-regulating Wnt/β-catenin activity. To test this possibility, we measured the level of Wnt/β-catenin signaling in 120-cell embryos (12 hpf) during the early stages of ANE restriction using the TCF-luciferase reporter plasmid, TopFlash [54]. Three different batches of embryos that had been injected with ΔFzl5/8 or treated with PKC inhibitor showed no significant difference in TopFlash activity when compared to controls (Figure 5E), suggesting that neither of these proteins affects early Wnt/β-catenin signaling. In contrast, TopFlash activity increased ∼2.5-fold on average in embryos lacking Fzl1/2/7 compared to controls (Figure 5E), indicating that signaling through Fzl1/2/7 negatively regulates Wnt/β-catenin activity in cleavage-stage embryos. Recently published experiments showed that introduction of mRNA encoding a dominant negative form of Fzl1/2/7 caused a *reduction* in TopFlash activity in cleavage-stage embryos and a loss of endoderm specification [55]. While this appears to conflict with our results, it is important to realize that interference with Fzl1/2/7 activity by misexpression of ΔFzl1/2/7 can interfere with the function of maternal Fzl1/2/7, whereas Fzl1/2/7 morpholino cannot. In keeping with this, embryos in which zygotic Fzl1/2/7 synthesis was blocked with a morpholino still expressed Wnt1 and Wnt8 (Figure 5F), whereas these are not expressed in embryos injected with ΔFzl1/2/7 [55]. Thus, in Fzl1/2/7 morphants, these Wnt ligands up-regulate Wnt/β-catenin- and Wnt/Fzl5/8-mediated ANE restriction, whereas the absence of these ligands in ΔFzl5/8-containing embryos leads to a reduction in Wnt/β-catenin activity. Collectively, these data suggest that Fzl1/2/7 signaling and PKC activity provide a buffer that limits the rate of ANE down-regulation by both of these Wnt signaling pathways (Figure 5G).

A possible concern in the ΔFz5/8 and ΔFz1/2/7 experiments is that elevating the levels of these proteins might influence the balance of signaling between the Wnt signaling pathways, for example, by competing for common components. To test this possibility, we overexpressed either wild-type Fzl5/8 or Fzl1/2/7 mRNA. In both cases, embryos developed normally and had normal *foxq2* expression patterns (Figure S6A). Next we showed that elevating the levels of Fzl1/2/7 mRNA did not change ΔFzl5/8's ability to prevent ANE restriction (Figure S6B) or prevent elimination of the ANE by excess Wnt1 mRNA (Figure S6C). Taken together these data indicate that the levels of endogenous Fzl receptors are not limiting. These data contrast with the Wnt1 and Wnt8 misexpression results, which showed that excess ligand can dramatically up-regulate ANE restriction (Figure 3Cb,d), suggesting that it is the levels of Wnt ligand in time and space and not those of the Wnt receptors that control the ANE restriction mechanism

### Dkk1 Antagonism of Wnt Signaling Protects the Final ANE Territory

**Figure 6:**
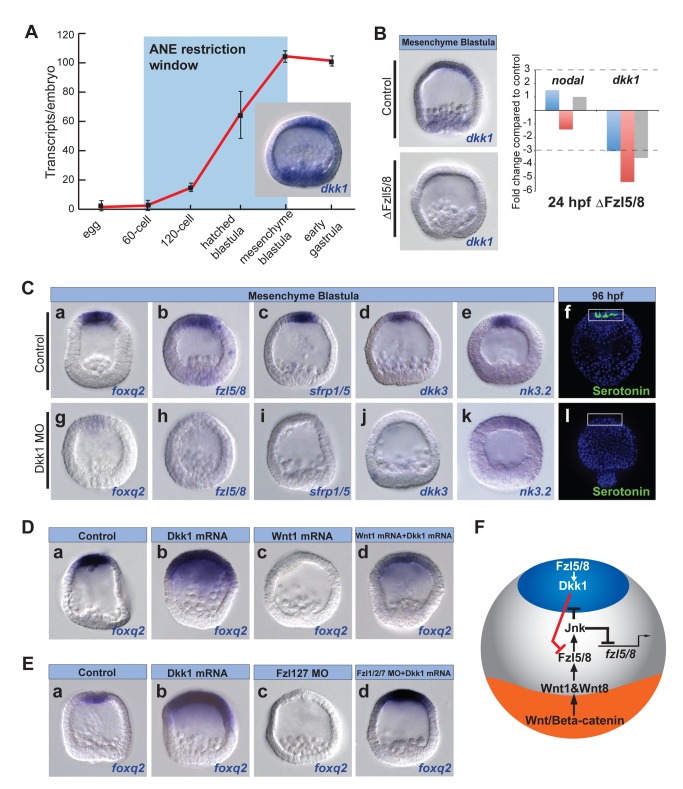
Dkk1 activity defines the ANE territory by antagonizing Wnt signaling. (A) qPCR measurements from three different batches of embryos showing the number of Dkk1 transcripts/embryo at the indicated stages during early development. Values were normalized to z12 transcripts (see Materials and Methods). (B) In situ hybridization (left) and qPCR (right) show that *dkk1* expression requires Fzl5/8 signaling (ΔFzl5/8 mRNA). (C) Six ANE markers show that ANE specification requires Dkk1. (D, E) Injected molecules are indicated at the top of panels. (D) Overexpression of Dkk1 prevents *foxq2* restriction to the anterior pole and suppression of *foxq2* expression by ectopic Wnt1. (E) Overexpression of Dkk1 rescues *foxq2* expression in embryos co-injected with Fzl1/2/7 morpholino. (F) Diagram showing a model for ANE restriction based on the data presented in this and previous figures. MO, morpholino.

Around the mesenchyme blastula stage (24 hpf), restriction of the ANE is complete and it constitutes a separate regulatory domain at the anterior end of the embryo with well-defined borders. Expression of *fzl5/8* is also restricted to this domain (Figure 2), raising the question of why Fzl5/8-mediated signaling does not continue to down-regulate the ANE regulatory state there. We hypothesized that the secreted Wnt antagonist, Dkk1, might play a role because, in most of the major clades, competition between anterior Wnt antagonism by Dkk1 and posterior Wnt signaling has been shown to regulate cell fates along the primary (AP) axis [8]. Very low-level *dkk1* expression was detectable as early as the 120-cell stage by qPCR (Figure 6A), and increased during the time of ANE restriction, reaching maximal levels by the mesenchyme blastula stage (24 hpf). At this time *dkk1* expression could be detected by in situ hybridization at the anterior end of the embryo as well as in a ring of cells surrounding the future site of gastrulation (Figure 6A, inset). Thus, *dkk1* was expressed at the right time and place to prevent anterior Wnt-mediated ANE down-regulation. Interestingly, expression of *dkk1* depended on Fzl5/8 signaling (Figure 6B), raising the possibility that Fzl5/8 signaling limits its own activity in anterior cells by promoting a negative feedback mechanism through this Wnt antagonist.

To test whether Dkk1 protects the ANE regulatory state from Wnt-mediated down-regulation, we monitored the expression of a set of genes encoding ANE regulatory factors in Dkk1 morphants at mesenchyme blastula stage by in situ hybridization. Each of the genes tested was severely down-regulated in these embryos (Figures 6Cg–k and S3H), and no serotonergic neurons developed in 4-d plutei (Figure 6Cl). Furthermore, overexpression of Dkk1 mRNA prevented restriction of *foxq2* expression (Figure 6Db) and rescued *foxq2* expression in embryos also overexpressing *wnt1* mRNA (88% rescue; *n* = 65) (Figure 6Dc versus d). Together these results indicate that Dkk1 can block the Wnt1/Fzl5/8-JNK-dependent ANE restriction mechanism. Interestingly, overexpression of Dkk1 also rescued *foxq2* expression in Fzl1/2/7 morphants (98% rescue; *n* = 64) (Figure 6Ec,Ed), suggesting that it may interfere with either Wntβ-catenin or Fzl5/8 signaling or both. There is some support for both possibilities. First, Dkk1 likely inhibits Fzl5/8 activity because the morphological phenotype (unpublished data) and *foxq2* expression pattern (cf., Figures 1F, 2Ag, and 6Db) of Dkk1 mRNA-injected embryos were more similar to those of embryos lacking functional Fzl5/8 than to those lacking Wnt/β-catenin signaling (cf., Figure 6Db and Figures 1H and 2Ag). Second, misexpressed Dkk1 can also interfere with endomesodermal gene expression, which depends on the Wnt/β-catenin pathway (Figure S4B).

## Discussion

**Figure 7:**
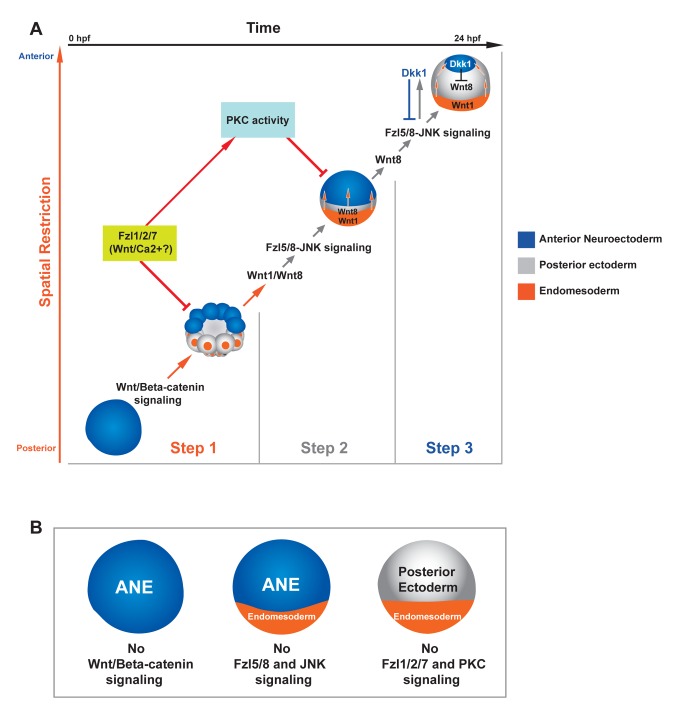
Three-step model for the balance of Wnt signaling interactions during ANE restriction. (A) The *y*-axis monitors the progress of spatial restriction of the ANE (blue), and the *x*-axis indicates the developmental timing of ANE restriction. Each image represents the position of the ANE in time and space. (Step 1) Initially, the maternal regulatory state in the absence of Wnt signaling supports ANE specification throughout the embryo. Then, nβ-catenin signaling in the posterior half of the embryo activates an unknown negative regulatory activity that blocks the accumulation of ANE factors, either by blocking their transcription directly or the activity of their ubiquitously expressed maternal activators. (Step 2) As development progresses, posterior nβ-catenin activates production of at least two Wnt ligands, Wnt1 and Wnt8, that are necessary to initiate the ANE restriction mechanism in posterior ectoderm beginning at the 60-cell stage. These secreted ligands signal through the Wnt receptor, Fzl5/8, activating the JNK pathway. The Wnt/JNK pathway progressively down-regulates expression of ANE factors during early blastula stages in all but the most anterior ectoderm. During Step 1 and possibly Step 2, Fzl1/2/7 signaling attenuates the nβ-catenin- and Fzl5/8-JNK-mediated down-regulation of ANE factors, preventing complete shutdown of ANE specification. PKC activity also antagonizes Fzl5/8-JNK-mediated ANE restriction, downstream of Fzl1/2/7 signaling. (Step 3) Expression of Wnt8, Wnt1, and/or another ligand X is activated in more anterior blastomeres. These ligands continue Fzl5/8-mediated ANE restriction until the late blastula-stage embryo activates production of the secreted Wnt antagonist, Dkk1, via Fzl5/8. Through negative feedback, Dkk1 limits Fzl5/8 activity, thereby defining the borders of the ANE. Orange arrows indicate the Wnt/β-catenin-mediated mechanism, gray arrows indicate the Wnt/JNK mediated mechanism, and red indicates Fzl1/2/7 and PKC interactions. (B) Diagram showing the state of the ANE in embryos lacking either Wnt/β-catenin, Fzl5/8 and pJNK, or Fzl1/2/7 and pPKC signaling.

The data presented here show that patterning the neuroectoderm along the AP axis of the early sea urchin embryo depends on an elegant spatiotemporal coordination and integration of the activities of three different Wnt signaling pathways. Throughout this process, a balance is achieved between the initial regulatory mechanisms that can specify the ANE ubiquitously, those that subsequently suppress it in posterior regions, and those that limit ANE suppression. The consequence is that ANE tissue is stably positioned only at the anterior pole of the embryo by the mesenchyme blastula stage. To summarize our current model (Figure 7), the first phase of ANE restriction requires Wnt/β-catenin and occurs very rapidly in posterior blastomeres by the 32- to 60-cell stage. Wnt/β-catenin signaling simultaneously activates expression of Wnt1 and Wnt8; these cells and these ligands initiate the second phase of ANE down-regulation in the posterior ectoderm (non-ANE ectoderm in the anterior hemisphere) by activating the Fzl5/8-JNK pathway, beginning around the 60-cell stage. As development progresses, Wnt1 and Wnt8 mRNAs accumulate in more anterior blastomeres, behind the front of ANE down-regulation. Whether these secreted ligands diffuse to the overlying ectoderm to directly activate Fzl5/8 or whether they act indirectly to stimulate production of other Wnt ligands that signal through this receptor is not known. Regardless, it is clear that Wnt1, Wnt8, Fzl5/8, and JNK are all required for full ANE down-regulation in the posterior ectoderm and suppression of transcription of *fzl5/8* itself. Clearly, Fzl5/8 plays a pivotal role in the ANE restriction process because it is necessary not only for the second phase of ANE restriction but also to stop that process in the third phase of ANE patterning when Fzl5/8 signaling leads to the expression of the Wnt receptor antagonist Dkk1 at the anterior pole. Thus, the coordination between the timing of auto-repression of *fzl5/8* transcription and activation of Dkk1 by Fzl5/8 ensures that this negative feedback loop reproducibly defines the ANE at the anterior pole of the embryo by mesenchyme blastula stage (24 hpf). The relative timing of Dkk1 production in the anterior ectoderm and ANE restriction in the rest of the embryo is critical and carefully controlled by a third Wnt pathway working through Fzl1/2/7 and PKC activities that limit Wnt/β-catenin and Wnt/JNK functions during the first two phases of ANE clearance.

Because all of these Wnt pathways affect the same developmental process (i.e., the specification of ANE versus non-ANE fates along the primary axis), they may function as components of an interactive Wnt signaling network rather than as separate pathways with different roles. Yet it appears that posterior Wnt/β-catenin and anterior Wnt/JNK signaling define two adjacent early regulatory domains in the sea urchin embryo. While our data suggest that these two signaling pathways activate different downstream regulatory programs in order to down-regulate ANE factors, both pathways are linked spatially and temporally by the activities of at least two common signaling components, Wnt1 and Wnt8 [49],[56]. These results are in keeping with recent evidence that individual Wnt ligands are able to activate distinct Wnt signaling branches, often in the same or adjacent territories [57]–[59]. However, it remains to be determined whether Wnt1 and/or Wnt8 act directly on cells in the anterior hemisphere in the ANE restriction process, although it is interesting that *wnt8* expression moves into posterior ectoderm cells as ANE factors move out. Alternatively, Wnt1 and Wnt8 may act indirectly by reinforcing the nβ-catenin gradient in the anterior-most cells of the posterior half of the embryo (i.e., near the equator), activating production of an unidentified intermediate Wnt ligand that is secreted from even more anterior cells and that activates Fzl5/8 signaling.

We found that the cardinal ANE regulatory genes, *six3* and *foxq2*, are not expressed in Fzl1/2/7 knockdowns. This unexpected phenotype is the exact opposite of the ANE expansion produced by interference with Wnt/β-catenin, Fzl5/8, and JNK signaling. The function of Fzl1/2/7 begins as early as the 32-cell stage, around the time that nβ-catenin is first detectable in posterior blastomeres [4] and at least 2 h before the ANE restriction process mediated by Fzl5/8 and JNK is observed. Since Fzl1/2/7 signaling significantly suppresses Wnt/β-catenin signaling during early cleavage stages, we propose that it reduces Wnt/β-catenin-dependent Fzl5/8 and JNK activities. This model suggests that Fzl1/2/7 signaling is essential for controlling the rate of progression of the ANE restriction mechanism along the AP axis, providing a “timing buffer” that prevents premature elimination of the ANE regulatory state during the early cleavage and blastula stages. We propose that one function of this Fzl1/2/7 “timing buffer” is to allow sufficient Fzl5/8-dependent accumulation of Dkk1 in the ANE by later blastula stages to protect it from Wnt signals and define its borders.

Since Fzl1/2/7 does not appear to signal through either the Wnt/JNK or the Wnt/β-catenin pathways during ANE restriction, we propose that it transduces signals through the Wnt/Ca^2+^ pathway. This mechanism may be similar to the situation in several other systems where Wnt/Ca^2+^ signaling affects early development either through the intracellular messengers CamKI, Calcineurin, and the transcription factor, NF-AT, or through PKC [60]. Similar to what we report here, the Wnt/Ca^2+^ pathway has been shown to antagonize Wnt/β-catenin signaling during vertebrate D/V axis specification [61],[62]. Interestingly, we found that blocking PKC activity with either an inhibitor or with Fzl1/2/7 morpholino had exactly the same effect on phosphorylated PKC levels and on the Fzl5/8-JNK-dependent re-specification of ANE to ectoderm fate. However, inhibiting the function of Fzl1/2/7 elevated Wnt/β-catenin activity, whereas loss of PKC activity did not. This result suggests that Fzl1/2/7 signaling activates two branches that affect ANE restriction, one that antagonizes early Wnt/β-catenin activity and another, mediated by pPKC, that blocks Fzl5/8-mediated ANE restriction in the anterior hemisphere. Thus, if Fzl1/2/7 mediates Wnt/Ca^2+^ signaling in the sea urchin embryo, it could affect several different downstream parallel pathways, any or all of which are necessary to prevent premature and complete elimination of the ANE regulatory state. Moreover, the involvement of Wnt/Ca^2+^ signaling in AP neuroectoderm patterning would be a first.

These considerations suggest that the function of Fzl1/2/7 in the early embryo is context-dependent, and we propose that the balance of information sent by this receptor through different Wnt signaling pathways is essential for correct specification and patterning. Recent data from several laboratories suggest that the same Fzl receptors can activate different Wnt signaling pathways, even in the same cells [50],[60],[63]. For example, the sea urchin Fzl1/2/7 homologue, Fz7, activates Wnt/β-catenin signaling and D/V axis specification in the early *Xenopus* embryo [63], but it also later activates Wnt/JNK and possibly the PKC signaling pathways that are required in the same general territory for convergent extension movements during gastrulation [57],[64],[65]. In the sea urchin embryo, our results and those of Lhomond et al. (2012) [55] are consistent with two early roles for Fzl1/2/7 – one stimulating endoderm specification via nβ-catenin through maternal Fzl1/2/7 in posterior blastomeres and another produced by zygotic Fzl1/2/7 that antagonizes early Wnt/β-catenin and subsequent Wnt/JNK signaling through an alternative Wnt pathway (Ca^+2^) that operates throughout the embryo. The balance between these pathways may favor Wnt/β-catenin signaling in the posterior half of the cleavage stage embryo because of localized Wnt/β-catenin pathway-specific co-factors in that part of the embryo [66],[67].

Striking parallels are emerging in the regulatory mechanisms that sea urchin and vertebrate embryos use to establish neural regulatory states at the anterior pole. Both embryos require Six3 for anterior neural development and share many homologous factors [24],[27]. Moreover, as shown here, in the absence of Wnt/β-catenin, and consequently of Nodal, BMP, and all other known signaling pathways, the regulatory state of *all* of the cells in the sea urchin embryo supports development of ANE from the very beginning of its specification. These data indicate that an initial ubiquitous maternal regulatory state activates ANE specification and that one of the most important roles of posterior Wnt/β-catenin signaling is to break the symmetry of this neural-promoting state. Similarly, in vertebrate embryos, an initial regulatory state is capable of activating ANE markers throughout the embryo in the absence of Wnt, Nodal, and BMP signaling [15],[20]–[22],[68]. Thus, this initial, broad activation of ANE specification, and its subsequent down-regulation, could be a widely shared property of deuterostome embryos.

**Figure 8:**
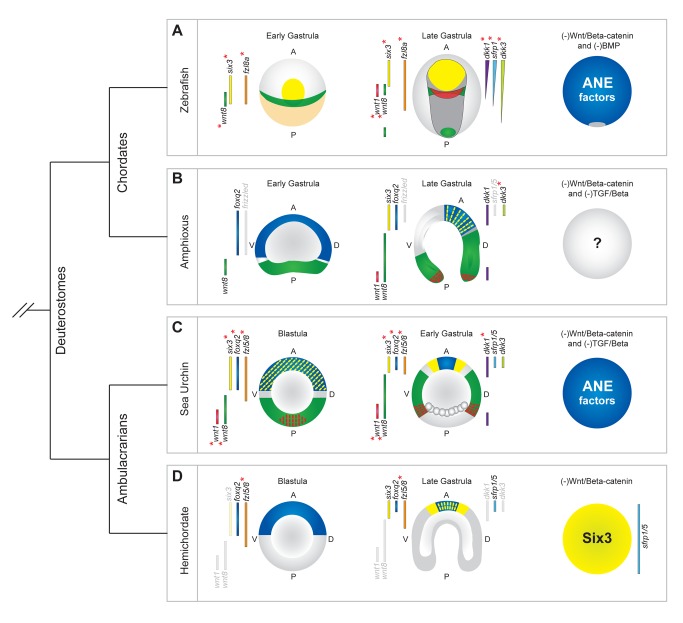
Conservation of expression patterns of orthologs of sea urchin genes that function in the development of ANE. The territorial expression of orthologs in each embryo is shown to the left and right of each diagram. In cases where there is no information concerning the expression pattern of a particular ortholog, it is represented in light gray. Red asterisks designate that functional studies show these factors are involved in AP patterning of the presumptive neuroectoderm. The images of embryos are colored to indicate the expression patterns of *foxq2*, *six3*, *wnt1*, and *wnt8*. (A) ANE factors are initially expressed throughout the presumptive zebrafish neuroectoderm at late blastula/early gastrula stages (left-hand diagram). These ANE factors are progressively down-regulated in posterior regions of the presumptive neuroectoderm (dark gray) during gastrulation by a mechanism involving Fzl8a, Wnt8, Wnt1, and Dkk1, until they are confined to the anterior pole (middle diagram). Factors such as sFrp1 and Dkk3 subsequently pattern the forebrain. The right-hand diagram shows that, in the absence of Wnt/β-catenin and BMP signaling, ANE factors are expressed throughout most of the embryo. Embryos are oriented with their dorsal sides facing up from the page. Data taken from [17],[21],[29],[73]–[77]. (B) In amphioxus embryos, *foxq2* is expressed throughout the anterior half, and *wnt8* throughout the vegetal plate, of early gastrula embryos (left-hand diagram). By late gastrula, *foxq2* and the putative ANE factors *dkk1*, *six3*, and *dkk3* are expressed in the anterior-most ectoderm. *wnt8* and *wnt1* are expressed posterior to these putative ANE factors, consistent with a role in the restriction of *foxq2*, *six3*, and *dkk3* expression to the anterior pole (middle diagram). Data taken from [78]–[82]. (C) Sea urchin embryo ANE factors are initially expressed throughout the presumptive ectoderm, and *wnt1* and *wnt8* are both expressed in the posterior half of blastula stage embryos (left-hand diagram). Then, ANE factors are progressively down-regulated from posterior ectoderm by a Wnt1, Wnt8, Fzl5/8, and Dkk1-dependent mechanism (middle diagram). In the absence of Wnt/β-catenin and TGF-β signaling, the entire sea urchin embryo expresses ANE factors (right-hand diagram). Data taken from this study and [22],[24]. (D) In blastula stage hemichordate embryos, *foxq2* is expressed broadly in the anterior half of the embryo (data show that *six3* and *fzl5/8* also are expressed broadly by early gastrula stages) (left-hand diagram). By late blastula stages, putative ANE factors *foxq2*, *six3*, and *sfrp1/5* are restricted to the anterior-most ectoderm, and functional data show that *sfrp1/5* restriction involves Fzl5/8 (middle diagram). In the absence of Wnt/β-catenin signaling the entire hemichordate embryo expresses putative ANE factors *six3* and *sfrp1/5* (right-hand diagram). Data taken from [2],[19],[83],[84].

The Wnt-dependent mechanism used for AP neuroectoderm patterning is still incompletely understood in vertebrates, in part because complex cell movements during patterning and the involvement of Wnt signaling in earlier specification events obscure the spatial and temporal relationships among the individual players [28],[30],[36]. In vertebrates, the only known Wnt pathway involved in the early restriction of ANE factors to the anterior pole is Wnt/β-catenin signaling [16]–[18]. Here, we show for the first time, to our knowledge, that the anterior Dkk1-posterior Wnt/β-catenin neuroectoderm patterning mechanism observed in vertebrates exists in a nonchordate deuterostome. These data strongly suggest the general Dkk1-Wnt/β-catenin AP patterning mechanism present in extant pre-bilaterian embryos was likely co-opted to pattern the neuroectoderm along the AP axis in the deuterostome ancestor. In addition to a posterior-to-anterior gradient of Wnt/β-catenin signaling, AP neuroectoderm patterning in the sea urchin embryo also requires Wnt/JNK signaling and an additional pathway mediated by Fzl1/2/7 that may function in Wnt/Ca^2+^ signaling. At present these are completely novel findings, but the fact that orthologs of several Wnt signaling components that function in these additional pathways in sea urchins (Fzl8, Wnt1, Wnt8, Dkk1) (Figure 8A,C) also are involved in posteriorizing the neural plate of vertebrate embryos (Figure 8A) [17],[31],[69] raises the possibility that this entire multistep mechanism was present in the common echinoderm/vertebrate ancestor and still operates to specify anterior neural identity in deuterostome embryos. Supporting this view, recent studies in hemichordates indicate that expression of homologues of sea urchin *foxq2*, *sfrp1/5*, and *six3* demarcate an anterior-most region of the embryo that is homologous to the vertebrate anterior neural ridge secondary patterning center [19]. Interestingly, these factors are initially broadly expressed and restricted to this region by an unknown mechanism that depends on posterior Wnt/β-catenin signaling and appears to require Fzl5/8 function in the anterior part of the embryo (Figure 8D). Moreover, there are similarities in the expression patterns of ANE genes (*dkk1*, *dkk3*, *six3*, *foxq2*) and those specifying endomesoderm (*wnt1* and *wnt8*) between the invertebrate chordate amphioxus and the sea urchin embryo (Figure 8B). For example, *foxq2* is initially expressed in a broad region and subsequently restricted to the anterior-most region. It can be completely cleared from this region of the embryo by LiCl treatment, which can elevate Wnt/β-catenin signaling [11], raising the possibility that amphioxus also uses the same ANE patterning mechanism. Thus, there is accumulating evidence that the ANE clearance mechanism described here may be used in a wide variety of deuterostomes. However, to date, only the work reported here reveals the intricate, interdependent Wnt signaling mechanisms that are required to confine the ANE regulatory state to the anterior end of the embryo.

## Materials and Methods

### Animals, Embryos, and Treatments

*Strongylocentrotus purpuratus* sea urchins were obtained from Point Loma Marine Invertebrate Lab, Lakeside, CA; The Cultured Abalone, Goleta, CA; or Marinus, Garden Grove, CA. Embryos were cultured in artificial seawater at 15°C. For drug treatments, eggs attached to a protamine sulfate-coated plate were fertilized in the presence of 2 mM 4-Aminobenzoic acid (PABA), and fertilization envelopes were removed by shear force. Treatments with the cell-permeable JNK Inhibitor 1, (L)-form, (EMD/Calbiochem) and the PKC inhibitor, Bisindolylmaleimide 1 (EMD/Calbiochem), were performed by diluting the stock solution to 50 µM or 3 µM, respectively. JNK Inhibitor 1, (L)-form is a specific inhibitor that blocks interactions between JNK and its transcriptional substrates, such as c-Jun and c-Fos, resulting in a knockout phenotype [46],[47]. Bisindolylmaleimide 1 is a selective inhibitor that specifically competes with the ATP binding site of most PKC isoforms [53]. As controls for the PKC inhibitor experiments, DMSO was added alone. These experiments were repeated with at least three different embryo batches, and each produced the same results.

### Preparation of cDNA Clones

The 24-h blastula total cDNA was used to obtain full-length clones for *dkk1*, *frizzled5/8*, *frizzled1/2/7*, *wnt1*, and a partial clone *of jnk* by PCR. The following primers were based on the sea urchin genome sequence: *Sp-dkk1* Forward 5′-AGA**ATG**GCGGCTCCTTCTGC-3′, Reverse 5′-TCATAATACAGTTAACTGGC-3′; *Sp-frizzled5/8* Forward 5′-AGA**ATG**GCTGCCTTCAGTGGAAC-3′, Reverse 5′-TCACACCTGTACATTTGGTA-3′; *Sp-frizzled1/2/7* Forward 5′-AGA**ATG**GGTTGGTTGGTGAGA-3′, Reverse 5′-TCATACATTGGCTGGTGCAC-3′; *Sp-wnt1* Forward 5′-AGA**ATG**AAACTTGAGTGGTTTG-3′, Reverse 5′-CTACAAGCATCTGTGCACG-3′; *Sp-jnk* Forward 5′-GAATGTGACGCATGCCAAGC-3′, Reverse 5′-GATCACCGCCGTCGTCTATTG-3′.

### mRNA and Morpholino Injections

Full-length *dkk1* and *wnt1 cDNA sequences* were inserted into pCS2+ vector for mis-expression studies. ΔFzl5/8-pCS2 and Wnt8-pCS2 were obtained from Jenifer Croce (CNRS/Villefranche sur Mer, France) and Christine Byrum (College of Charleston, Charleston, SC), respectively. pCS2 constructs were linearized with Not1 and mRNA was synthesized with mMessage Machine kit (Ambion), purified by LiCl precipitation and ∼20 pl injected at the following concentrations: Fzl1/2/7 mRNA = 1.0–1.5 µg/µL; Fzl5/8 mRNA = 2.0 µg/µL; ΔFzl5/8 mRNA = 2.0 µg/µL; Wnt1 mRNA = 0.01–0.1 µg/µL; Wnt8 mRNA = 0.5–1.0 µg/µL; Dkk1 mRNA = 3.0 µg/µL; Axin mRNA = 1.0 µg/µL; Tcf-Eng mRNA = 0.5–1.0 µg/µL.

*S. purpuratus* EST sequences for *wnt1*, *fzl1/2/7*, and *fzl5/8* as well as sequence information from 5′ RACE on *dkk1* were used to generate translation-blocking morpholino oligonucleotides. A splice-blocking morpholino oligonucleotide was designed for the second exon–intron boundary of *wnt8*, which produces transcripts encoding a protein lacking sequence from the second exon, which was verified by PCR (Figure S2A). The morpholinos were produced by Gene-Tools LLC (Eugene, OR). The sequences and injection concentrations were: Wnt1 MO1: 5′-ACGCTACAAACCACTCAAGTTTCAT-3′ (1.8 mM); Wnt1 MO2: 5′-ATCCTCATCAAAACTAACTCCAAGA-3′ (0.4 mM); Wnt8 splice MO: 5′-GTAAAGTGTTTTTCTTACCTTGGAT-3′ (0.7 mM); Wnt8 MO2: 5′-GTACACTCCAATAAAAGAAATCAAA-3′ (0.6 mM) [49]; Fzl1/2/7 MO1: 5′-CATCTTCTAACCGTATATCTTCTGC-3′ (1.3 mM); Fzl1/2/7 MO2: 5′-ACAGATCTCCTTTAACAAGGGTAGA-3′ (2.2 mM); Fzl5/8 MO1: 5′-GGATTGTTCCACTGAAGGCAGCCAT-3′ (2.25 mM); Fzl5/8 MO2: 5′-ATGTTTATGGTCTGATGGCAATCGC-3′ (0.6 mM); Dkk1 MO1: 5′-GCGTCTAAATCCTAAATTCCTTCCT-3′ (1.5–1.6 mM); Dkk1 MO2: 5′-ATCGTTGGTAGTTGCAGAAATTCGT-3′ (0.7–0.85 mM); and JNK splice MO: 5′-CCTCATCGTTCTAGACTCACCGTTC-3′ (1.0–1.25 mM).

Embryos were injected immediately after fertilization with solutions containing FITC, 20% glycerol, and mRNA and/or morpholino oligonucleotides. All injected embryos were cultured at 15°C. Microinjection experiments were performed using at least three different batches of embryos, and each experiment consisted of 50–150 embryos unless otherwise stated. Experiments were scored only if a change in phenotype or marker expression was seen in at least 85%–90% of the manipulated embryos.

### Quantitative PCR (qPCR)

qPCR was performed as described previously [24]. Each experiment was repeated with embryos from at least three different mating pairs, and each PCR reaction was carried out in triplicate. The primer set information can be found in Table S1. For developmental expression analysis, the number of transcripts per embryo was estimated based on the Ct value of the z12 transcript [70].

### Whole-Mount in Situ Hybridization

The probes for each gene analyzed correspond to the full-length cDNA sequence. Alkaline phosphatase and three-color fluorescent in situ hybridization were carried out as previously described [24],[71]. For the three-color in situ hybridization, *foxq2* was labeled with fluorescein and detected with Cy5-TSA, *wnt1* was labeled with DNP and detected with Cy3-TSA, and *wnt8* was labeled with DIG and detected with fluorescein-TSA.

### Immunohistochemistry

Embryos were fixed in 2%–4% paraformaldehyde in artificial seawater at RT for 20 min and washed 5 times in phosphate-buffered saline containing 0.1% Tween-20. Embryos were incubated with primary antibodies at 4°C overnight at a dilution of 1∶1,000 for serotonin (Sigma, St. Louis, MO) and synaptotagminB/1e11 [72]. Primary antibodies were detected by incubating embryos with Alexa-coupled secondary antibodies for 1 h at RT. Nuclei were stained with DAPI.

### Western Analysis

Protein extracts were prepared by adding 30 µL of lysis buffer (25 mM Tris-HCL pH 7.4; 150 mM NaCl; 5 mM EDTA; with PhosSTOP phosphatase and Complete Mini protease inhibitor cocktails; Roche, Indianapolis, IN) to a pellet of 300 injected embryos. Embryos were crushed 4–5 times with a pestle, immediately spun at 16,000 RCF for 15 min at 4°C, and the supernatant was stored at −80°C until use. Samples were thawed on ice and 4× NuPage Running Buffer containing 4% SDS and 10% 2-ME was added. Samples were heated at 80°C for 3–5 min and 20 µL of each sample was run on 4%–12% NuPage Bis-Tris gradient gel (Invitrogen, Grand Island, NY), transferred to nitrocellulose. Membranes were probed overnight at 4°C in Phosphate Buffered Saline+0.1% Tween-20 (PBST)+3% BSA with a poly-clonal Phospho-PKC(pan) (β11 Ser660) antibody (Cell Signaling Technology, Danvers, MA) (1∶250) that recognizes a region that includes serine 660 and detects endogenous levels of phosphorylated PKC α, β1, β11, δ, ε, η, and θ. The recognition sequence is conserved in *S. purpuratus* PKC isoforms. Membranes were washed 3–5 times in room temperature PBST and probed for 1 h at room temperature in PBST+3% Bovine Serum Albumin with an enhanced chemiluminescent anti-rabbit IgG horseradish peroxidase secondary antibody (GE Healthcare, Piscataway, NJ). After 3–5 more washes in PBST, the membranes were developed and imaged.

### Luciferase Assays

Promega Dual Luciferase Reporter System (Promega) was used to perform dual luciferase assays. Embryos (350–400) were injected with linearized TopFlash-Firefly Luciferase (REF) and Endo16-Renilla Luciferase plasmids at concentrations of 20 ng/µL and 10 ng/µL, respectively, along with 10 ng/µL of linearized genomic DNA carrier. The Firefly and Renilla luciferase signals were recorded with a plate style luminometer using Promega's suggested protocol. The level of luciferase activity was normalized to the level of Renilla activity for each experiment. All experiments were repeated three times using separate batches of embryos.

## Supporting Information

Figure S1 TCF-Eng and Axin mRNA overexpression block Wnt/β-catenin-dependent specification of endomesoderm and patterning of the ANE. (Ae, Af, Ag) TCF-Eng mRNA overexpression prevents restriction of *foxq2* expression at early cleavage and blastula stages, related to Figure 1. (Ad, Ah) Serotonergic neurons are expressed throughout embryos injected with TCF-Eng mRNA. (Ba–c, Be–g) Axin mRNA overexpression prevents expression of three key endomesoderm regulatory factors, *blimp1b*, *hox11/13b*, and *gcm*. (Bd, Bh) Axin injected embryos develop into dauer blastulae.
(TIF)

Figure S2 Expression of *fzl5/8*, *fzl1/2/7*, *fzl9/10*, and *fzl4* during early development of the sea urchin embryo, related to Figures 2 and 4. (A) qPCR measurements from three different cultures of embryos showing the approximate number of *fzl5/8*, *fzl1/2/7*, *fzl9/10*, and *fzl4* transcripts per embryo at 0 hpf, 60-cell, 120-cell, hatched blastula, and mesenchyme blastula stages. The *y*-axis shows the approximate number of transcripts per embryo based on the Ct value of *z12* transcripts, whose absolute concentrations are known at each stage (Wang et al., 2004) [70]. (B) Whole mount in situ hybridization for *fzl1/2/7*, *fzl5/8*, and *foxq2* mRNAs during ANE restriction. (Ba–d) *fzl1/2/7* expression. (Be–h) *fzl5/8* expression. (Bi–l) *foxq2* expression. All samples were examined at the stages indicated above each column. (C) Whole mount in situ hybridization showing that JNK is ubiquitously expressed during the ANE restriction process (60-cell to mesenchyme blastula stage).
(TIF)

Figure S3 Additional morpholino and inhibitor phenotypes, related to Figures 2–6. (A–E) The ANE is expanded in embryos injected with JNK-MO1 (A), Wnt1-MO2 (B), Wnt8-MO2 (Wikramanayake et al., 2004) [49] (C), Fzl5/8-MO1 (D), and Fzl5/8-MO2 (E). (F) DIC images of 90-hpf embryos injected with Fzl1/2/7-MO1 and Dkk-MO1. Arrowheads indicate location of thickened columnar epithelium corresponding to the ANE in normal embryos or lack of this epithelium in Fzl1/2/7 morphants. (G, H) ANE factors are severely down-regulated in embryos injected with Fzl1/2/7-MO2 (G) and Dkk1-MO2 (H). (Ia–e) Embryos treated with the PKC inhibitor, Bisindolylmaleimide 1, lack serotonergic neurons and a complete skeleton, but have a dorsal-ventral axis and have undergone gastrulation. (J and K) Controls for the efficacy of the Wnt8 and JNK splice-blocking morpholinos. PCR analysis of control glycerol-injected and embryos injected with a Wnt8 splice-blocking morpholino (Wnt8-MO1 in methods) (Ja) or JNK splice-blocking morpholino (JNK-MO1 in methods) (Ka). Expected control PCR product size for *wnt8* = 357 bp; expected Wnt8-MO1 PCR product size for *wnt8* = 315 bp; expected control PCR product size for *jnk* = 690 bp; expected JNK splice MO PCR product size for *jnk = *530 bp. Diagrams of the intron-exon organization of *wnt8* (Jb) and *jnk* (Kb) pre-mRNAs. Primers used to characterize the mRNA products in (Ia) and (Ja) (arrows). Position of the target sequence for the morpholino (red bar). JNK catalytic domains is in the deleted exon (blue bar) (Kb). MO, morpholino.
(TIF)

Figure S4 Expression of *wnt1* during early development and effects of perturbing Wnt signaling via Dkk1, Axin mis-expression, and a Wnt1 morpholino, related to Figures 3 and 6. (A) Whole mount in situ hybridization for *wnt1* during ANE restriction. (B) Overexpression of Dkk1 blocks the expression of the endomesoderm marker *z13*; (C) overexpression of Axin blocks expression of *wnt1*; (D) *wnt8* expression is not regulated by Wnt1 signaling; (E) *wnt1* expression does not depend on Wnt8 signaling.
(TIF)

Figure S5 Phenotypes produced are severely vegetalized/posteriorized by Wnt1 misexpression, require pJNK and Fz5/8, and can be antagonized by Dkk1. (A) pJNK is necessary for full down-regulation of the ANE by Wnt1. Wnt1 mRNA overexpression eliminates *foxq2* expression (Aa, b), related to Figure 3. Addition of JNK(−) inhibitor rescues *foxq2* expression in more than half the embryos (percentages of embryos with different *foxq2* mRNA levels are given in the upper right; Ac–e). (B) Fzl5/8 is necessary for and Dkk1 antagonizes posteriorization by Wnt1 signaling; related to Figures 3 and 6. DIC images of 72 hpf pluteus embryos. (a) Control embryos. (b) Embryos misexpressing *wnt1* have a severe vegetalized/posteriorized phenotype. (c) Embryos misexpressing both *wnt1* and *dkk1* have a normal phenotype. (d) Embryos misexpressing *wnt1* and Δ*fzl5/8* have the Δ*fzl5/8* phenotype.
(TIF)

Figure S6 The expression level of Fzl receptors is not a rate-limiting step that influences the balance of Wnt signaling in embryos during ANE restriction, related to Figures 2, 3, and 5. (A) Overexpression of wild-type Fzl5/8 (b) or Fzl1/2/7 (c) does not perturb the ANE restriction mechanism. (B) Elevated level of Fzl1/2/7 mRNA does not reduce ΔFzl5/8-mediated ANE restriction. (C) Elimination of the ANE by excess Wnt1 mRNA is not perturbed by overexpression of WT Fzl1/2/7 mRNA.
(TIF)

Table S1 qPCR primer pairs used for expression analysis.
(DOCX)

## Abbreviations

Δ: dominant negative
ANE: anterior neuroectoderm
AP: anterior–posterior
Dkk: Dickkopf
Fzl: Frizzled
hpf: hours postfertilization
JNK: c-Jun N-terminal Kinase
MO: morpholino oligonucleotide
nβ-catenin: nuclear β-catenin
PKC: Protein Kinase C
qPCR: real-time quantitative PCR
Tcf-Eng: Tcf-Engrailed
