# Supplementary figures and images for: Integration of Canonical and Noncanonical Wnt Signaling Pathways Patterns the Neuroectoderm Along the Anterior–Posterior Axis of Sea Urchin Embryos
Source: PLoS Biol. 2013 Jan 15;11(1):e1001467. doi: 10.1371/journal.pbio.1001467 (PMC3545869; doi:10.1371/journal.pbio.1001467)

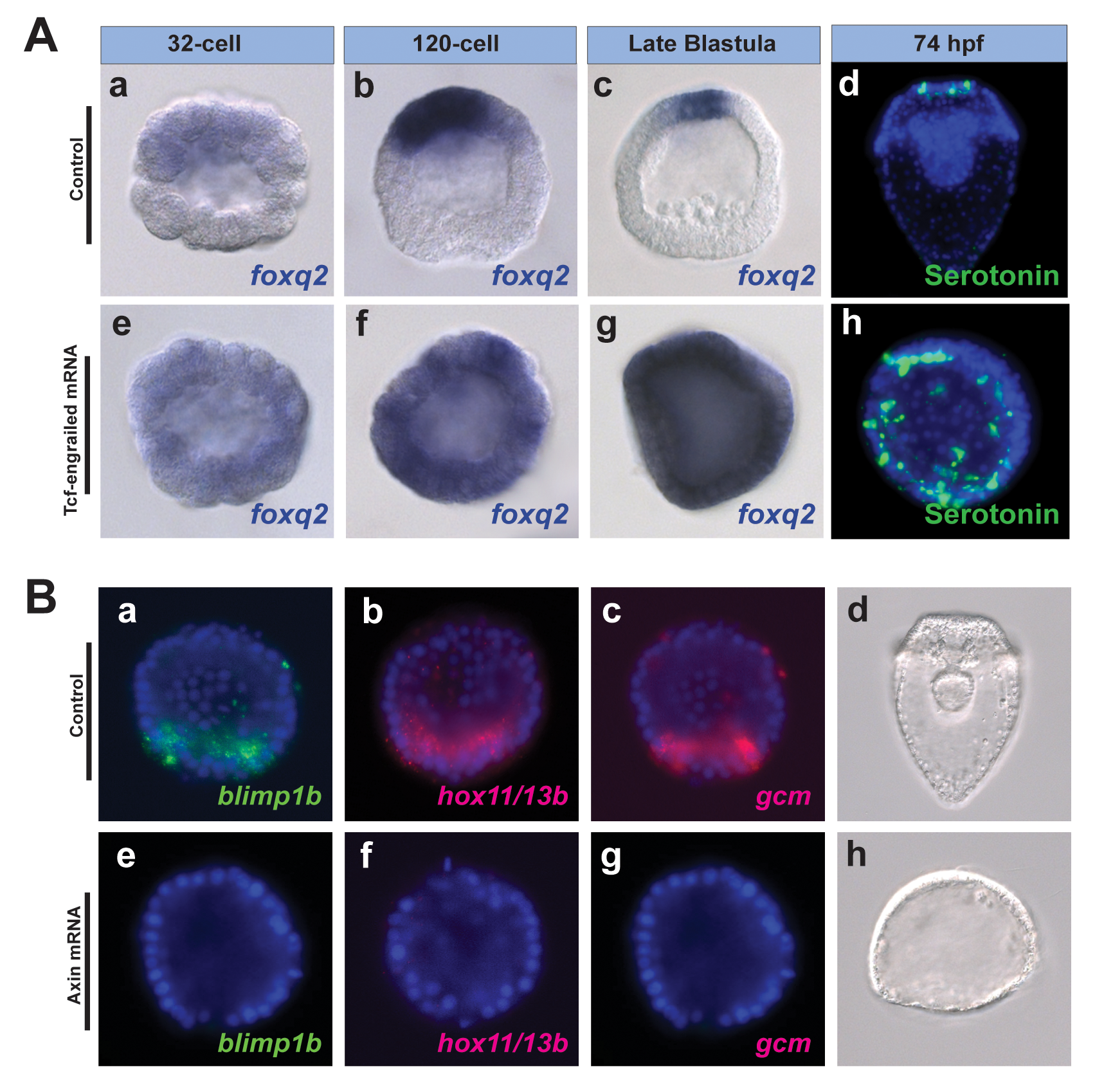

Supplement: Figure S1 — TCF-Eng and Axin mRNA overexpression block Wnt/β-catenin-dependent specification of endomesoderm and patterning of the ANE. (Ae, Af, Ag) TCF-Eng mRNA overexpression prevents restriction of foxq2 expression at early cleavage and blastula stages, related to Figure 1. (Ad, Ah) Serotonergic neurons are expressed throughout embryos injected with TCF-Eng mRNA. (Ba–c, Be–g) Axin mRNA overexpression prevents expression of three key endomesoderm regulatory factors, blimp1b, hox11/13b, and gcm. (Bd, Bh) Axin injected embryos develop into dauer blastulae. (TIF) [file pbio.1001467.s001.tif]

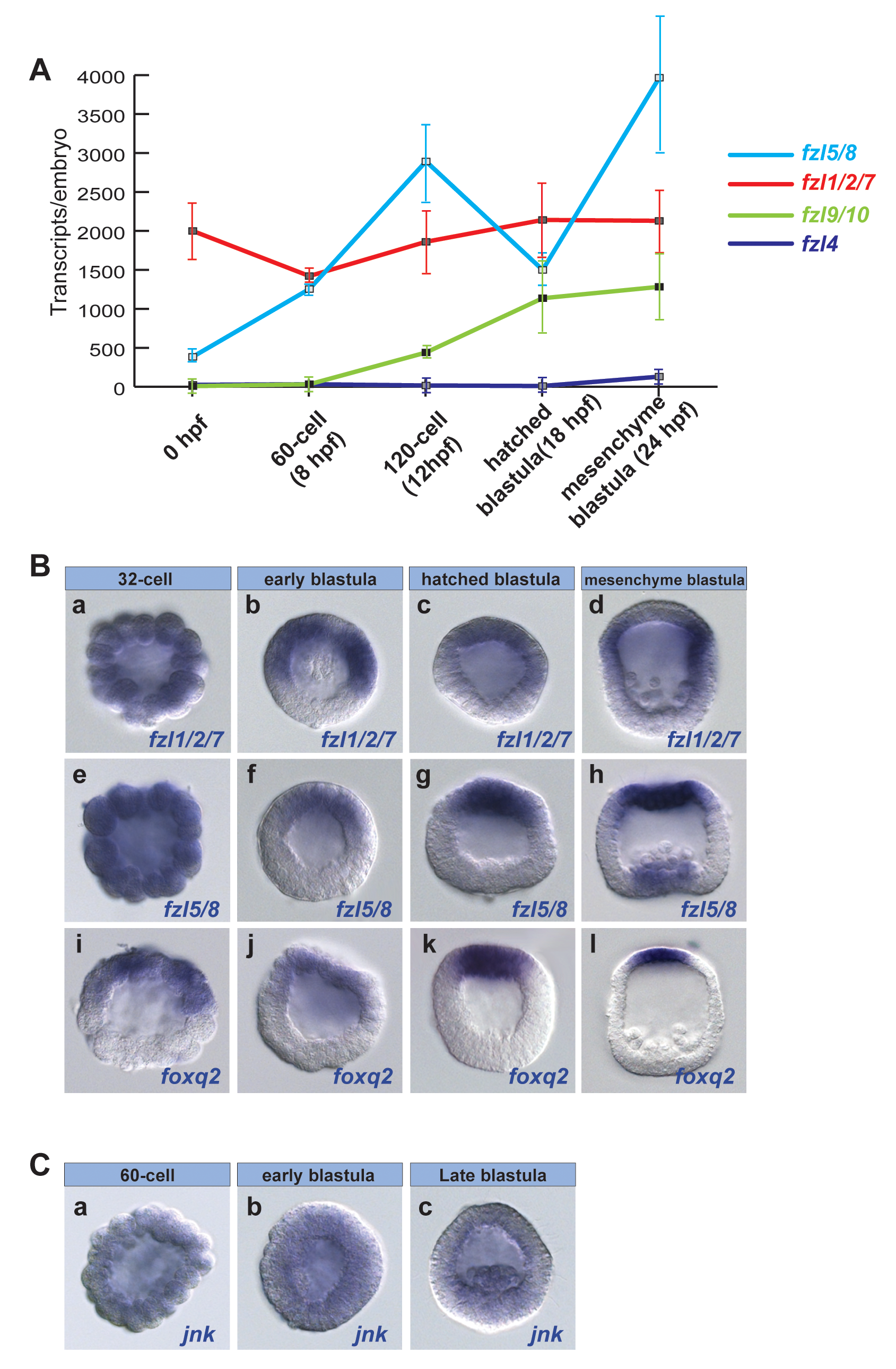

Supplement: Figure S2 — Expression of fzl5/8, fzl1/2/7, fzl9/10, and fzl4 during early development of the sea urchin embryo, related to Figures 2 and 4. (A) qPCR measurements from three different cultures of embryos showing the approximate number of fzl5/8, fzl1/2/7, fzl9/10, and fzl4 transcripts per embryo at 0 hpf, 60-cell, 120-cell, hatched blastula, and mesenchyme blastula stages. The y-axis shows the approximate number of transcripts per embryo based on the Ct value of z12 transcripts, whose absolute concentrations are known at each stage (Wang et al., 2004) [70]. (B) Whole mount in situ hybridization for fzl1/2/7, fzl5/8, and foxq2 mRNAs during ANE restriction. (Ba–d) fzl1/2/7 expression. (Be–h) fzl5/8 expression. (Bi–l) foxq2 expression. All samples were examined at the stages indicated above each column. (C) Whole mount in situ hybridization showing that JNK is ubiquitously expressed during the ANE restriction process (60-cell to mesenchyme blastula stage). (TIF) [file pbio.1001467.s002.tif]

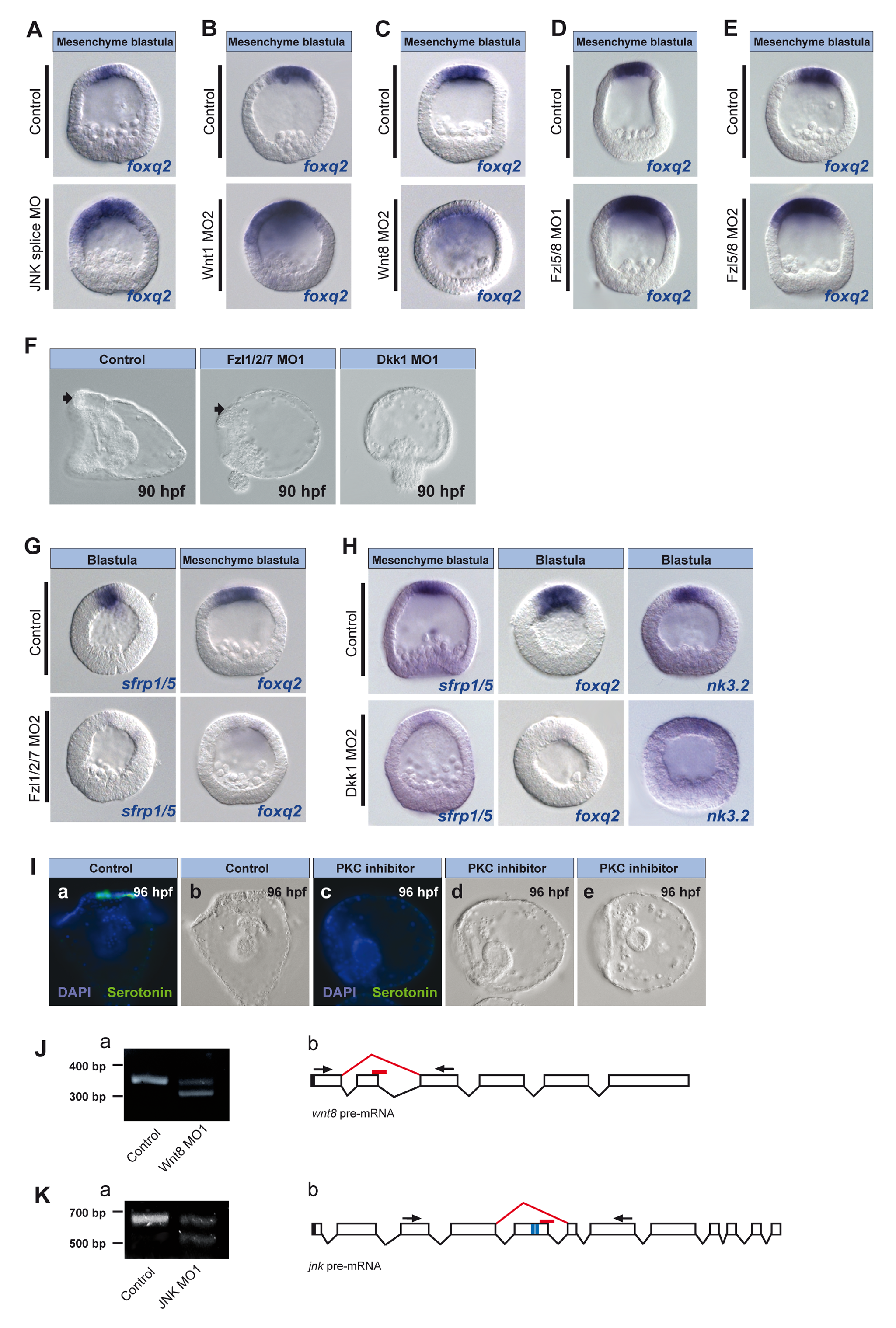

Supplement: Figure S3 — Additional morpholino and inhibitor phenotypes, related to Figures 2–6. (A–E) The ANE is expanded in embryos injected with JNK-MO1 (A), Wnt1-MO2 (B), Wnt8-MO2 (Wikramanayake et al., 2004) [49] (C), Fzl5/8-MO1 (D), and Fzl5/8-MO2 (E). (F) DIC images of 90-hpf embryos injected with Fzl1/2/7-MO1 and Dkk-MO1. Arrowheads indicate location of thickened columnar epithelium corresponding to the ANE in normal embryos or lack of this epithelium in Fzl1/2/7 morphants. (G, H) ANE factors are severely down-regulated in embryos injected with Fzl1/2/7-MO2 (G) and Dkk1-MO2 (H). (Ia–e) Embryos treated with the PKC inhibitor, Bisindolylmaleimide 1, lack serotonergic neurons and a complete skeleton, but have a dorsal-ventral axis and have undergone gastrulation. (J and K) Controls for the efficacy of the Wnt8 and JNK splice-blocking morpholinos. PCR analysis of control glycerol-injected and embryos injected with a Wnt8 splice-blocking morpholino (Wnt8-MO1 in methods) (Ja) or JNK splice-blocking morpholino (JNK-MO1 in methods) (Ka). Expected control PCR product size for wnt8 = 357 bp; expected Wnt8-MO1 PCR product size for wnt8 = 315 bp; expected control PCR product size for jnk = 690 bp; expected JNK splice MO PCR product size for jnk = 530 bp. Diagrams of the intron-exon organization of wnt8 (Jb) and jnk (Kb) pre-mRNAs. Primers used to characterize the mRNA products in (Ia) and (Ja) (arrows). Position of the target sequence for the morpholino (red bar). JNK catalytic domains is in the deleted exon (blue bar) (Kb). MO, morpholino. (TIF) [file pbio.1001467.s003.tif]

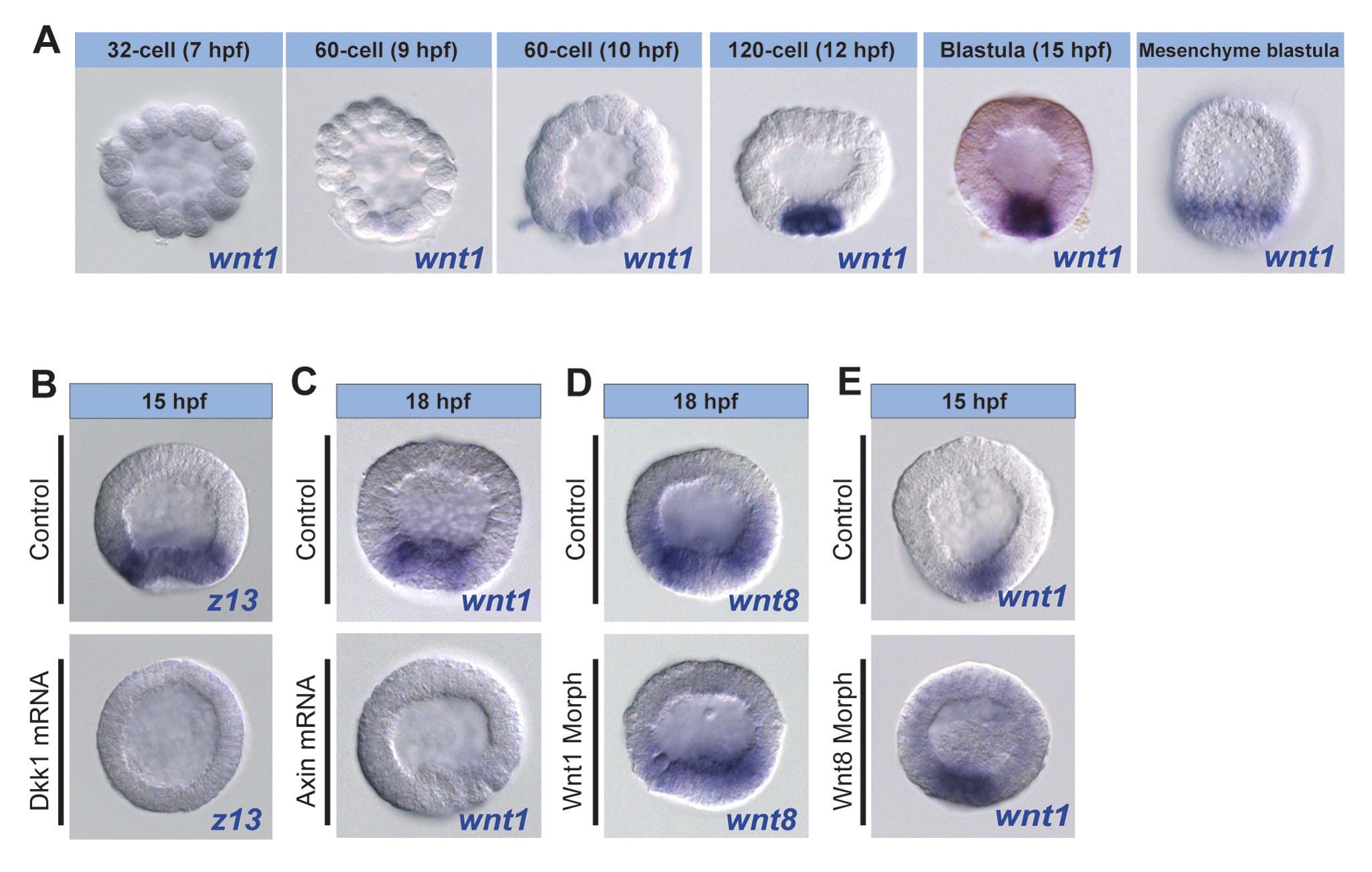

Supplement: Figure S4 — Expression of wnt1 during early development and effects of perturbing Wnt signaling via Dkk1, Axin mis-expression, and a Wnt1 morpholino, related to Figures 3 and 6. (A) Whole mount in situ hybridization for wnt1 during ANE restriction. (B) Overexpression of Dkk1 blocks the expression of the endomesoderm marker z13; (C) overexpression of Axin blocks expression of wnt1; (D) wnt8 expression is not regulated by Wnt1 signaling; (E) wnt1 expression does not depend on Wnt8 signaling. (TIF) [file pbio.1001467.s004.tif]

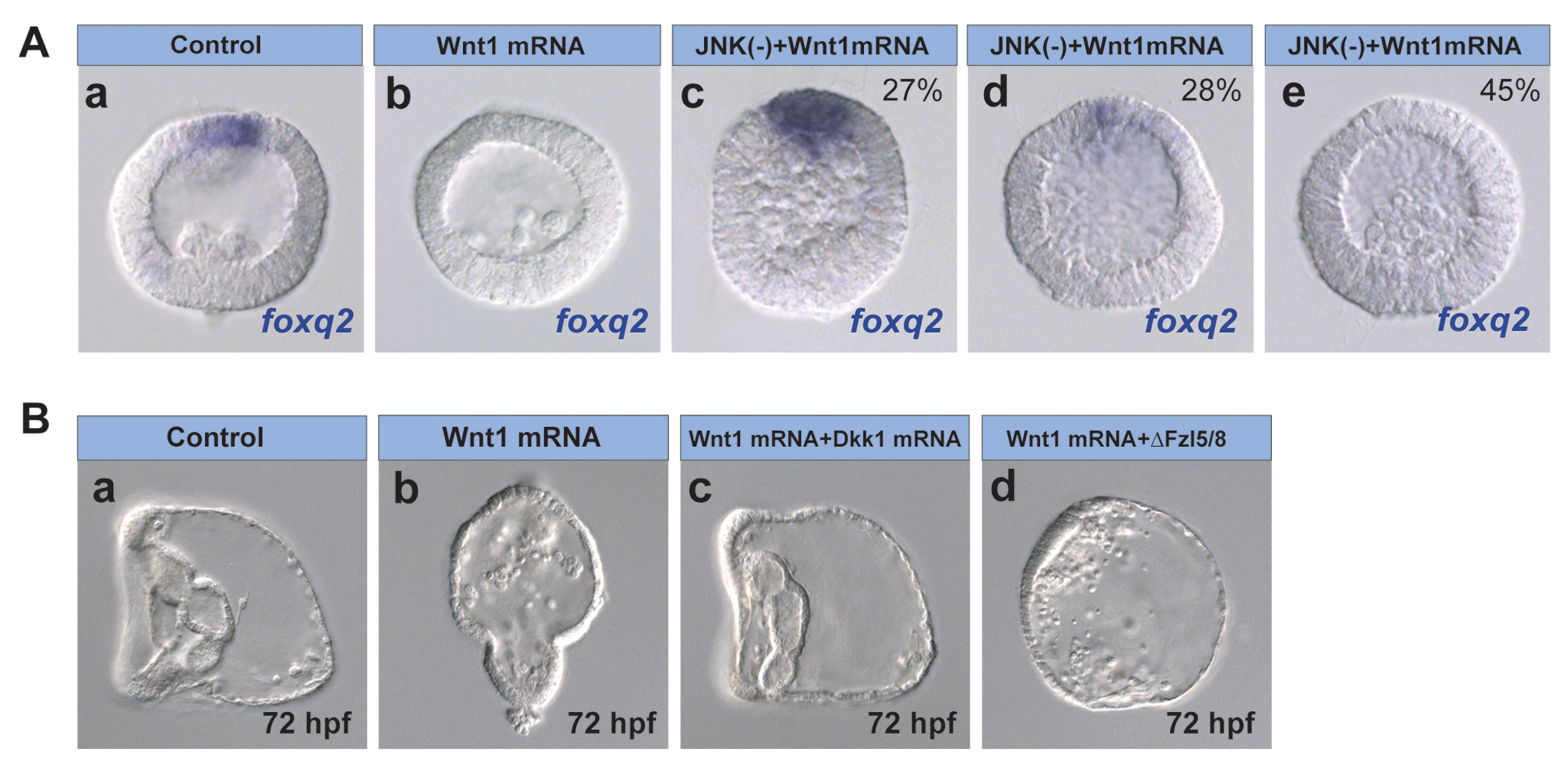

Supplement: Figure S5 — Phenotypes produced are severely vegetalized/posteriorized by Wnt1 misexpression, require pJNK and Fz5/8, and can be antagonized by Dkk1. (A) pJNK is necessary for full down-regulation of the ANE by Wnt1. Wnt1 mRNA overexpression eliminates foxq2 expression (Aa, b), related to Figure 3. Addition of JNK(−) inhibitor rescues foxq2 expression in more than half the embryos (percentages of embryos with different foxq2 mRNA levels are given in the upper right; Ac–e). (B) Fzl5/8 is necessary for and Dkk1 antagonizes posteriorization by Wnt1 signaling; related to Figures 3 and 6. DIC images of 72 hpf pluteus embryos. (a) Control embryos. (b) Embryos misexpressing wnt1 have a severe vegetalized/posteriorized phenotype. (c) Embryos misexpressing both wnt1 and dkk1 have a normal phenotype. (d) Embryos misexpressing wnt1 and Δfzl5/8 have the Δfzl5/8 phenotype. (TIF) [file pbio.1001467.s005.tif]

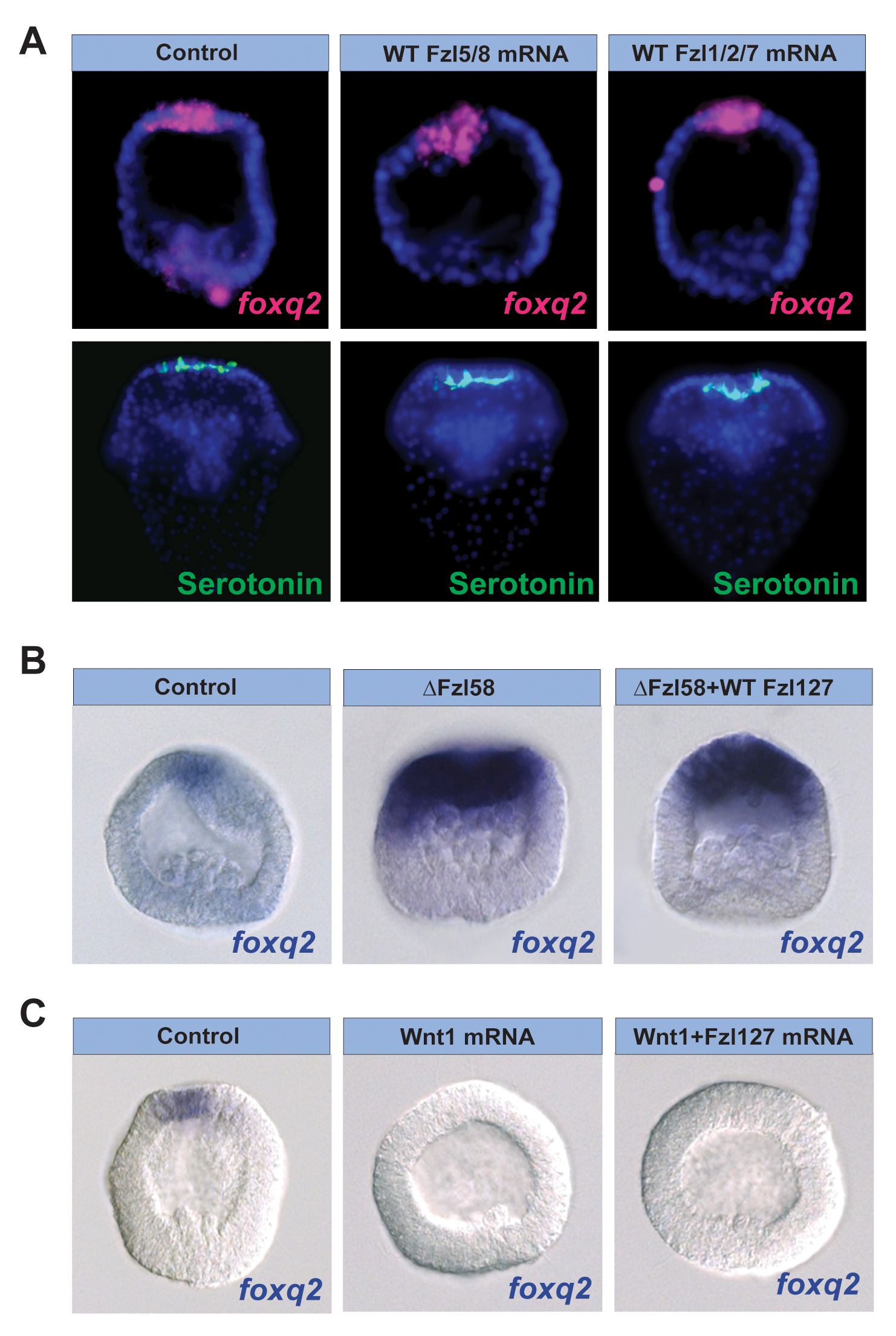

Supplement: Figure S6 — The expression level of Fzl receptors is not a rate-limiting step that influences the balance of Wnt signaling in embryos during ANE restriction, related to Figures 2, 3, and 5. (A) Overexpression of wild-type Fzl5/8 (b) or Fzl1/2/7 (c) does not perturb the ANE restriction mechanism. (B) Elevated level of Fzl1/2/7 mRNA does not reduce ΔFzl5/8-mediated ANE restriction. (C) Elimination of the ANE by excess Wnt1 mRNA is not perturbed by overexpression of WT Fzl1/2/7 mRNA. (TIF) [file pbio.1001467.s006.tif]
