# Supplementary material for: Integration of Canonical and Noncanonical Wnt Signaling Pathways Patterns the Neuroectoderm Along the Anterior–Posterior Axis of Sea Urchin Embryos
Source: PLoS Biol. 2013 Jan 15;11(1):e1001467. doi: 10.1371/journal.pbio.1001467 (PMC3545869; doi:10.1371/journal.pbio.1001467)
Supplement: Table S1 — qPCR primer pairs used for expression analysis. (DOCX) [file pbio.1001467.s007.docx]

**Table S1**

*Sp-mitochondrial* Forward 5’-ACTCTCTCCTCGGAGCTATA-3’

*12s rRNA* Reverse 5’-GTATAATTTTTGCGTATTCGGC-3’

*Sp-zic2* Forward 5’-GAGGGATGTGATCGTCGTTT-3’

Reverse 5’-ACTGCTGTCGTTGGCTTCTT-3’

*Sp-foxQ2* Forward 5’-TAACGAAAGGAGCTGGAGGA-3’

Reverse 5’-GCTTGCCGTCTCCTGTAATC-3’

*Sp-fgfr-like* Forward 5’-CATAACAGAGGGCAGGCAAT­-3’

Reverse 5’-ATGTTGTGCCTCCAAACTCC-3’

*Sp-dkk1* Forward 5’-GTGTTCGCAAGGTCTCTC-3’

Reverse 5’-GTCGTTCTTGCTCGGAAG-3’

*Sp-hbn* Forward 5’-CTCGAGTCCCAGTGGGTTTA-3’

Reverse 5’-TCCCGATTGGATTTCTCAAG-3’

*Sp-sfrp1/5* Forward 5’-CTGGCCAGCTATGCTTCAGTG-3’

­ Reverse 5’-CCCAATGTACTTCTTGCCGTCT-3’

*Sp-dkk3* Forward 5’-ATGGTTCGGATTATGGACACCGT-3’

Reverse 5’-CTGGGATGTTCTCTTTCCAGATC-3’

*Sp-six3* Forward 5’-CAGAGCTGTCGTTTCCTTCC-3’

Reverse 5’-GGGCCTTCCTCTGAGTTTCT-3’

*Sp-nodal* Forward 5’-GAATCGGCTGGGACGAATGG-3’

Reverse 5’-CGTGGTTGCTTGGGTTGTCG-3’

*Sp-fzl1/2/7* Forward 5’-CCGGGTCCAGACTATCTTGT-3’

Reverse 5’-AAAACTGGGTCCACGATCTC-3’

*Sp-fz4* Forward 5’-CAAGCGGAATGTGGATAATG-3’

Reverse 5’-TTCTGTCACGTCCGTTCTTC-3’

*Sp-fz5/8* Forward 5’-TGTACCGATGTGTCGTGATG-3’

Reverse 5’-CCGGAGAACATTGGATCTCT-3’

*Sp-fz9/10* Forward 5’-CAACGTGGAGCCTTATCCTT-3’

Reverse 5’-GCAATGTCCCTGTGTGTTTC-3’

*Sp-z12*  Forward 5’-AGTCGTCCAGCCATGTCTTT-3’

Reverse 5’-AAGCACACCTCGCACCTATC-3’
